# Genetic Changes to a Transcriptional Silencer Element Confers Phenotypic Diversity within and between *Drosophila* Species

**DOI:** 10.1371/journal.pgen.1005279

**Published:** 2015-06-26

**Authors:** Winslow C. Johnson, Alison J. Ordway, Masayoshi Watada, Jonathan N. Pruitt, Thomas M. Williams, Mark Rebeiz

**Affiliations:** 1 Department of Biological Sciences, University of Pittsburgh, Pittsburgh, Pennsylvania, United States of America; 2 Department of Biology, Faculty of Science, Ehime University, Matsuyama, Japan; 3 Department of Biology, University of Dayton, Dayton, Ohio, United States of America; New York University, UNITED STATES

## Abstract

The modification of transcriptional regulation has become increasingly appreciated as a major contributor to morphological evolution. However, the role of negative-acting control elements (e.g. silencers) in generating morphological diversity has been generally overlooked relative to positive-acting “enhancer” elements. The highly variable body coloration patterns among Drosophilid insects represents a powerful model system in which the molecular alterations that underlie phenotypic diversity can be defined. In a survey of pigment phenotypes among geographically disparate Japanese populations of *Drosophila auraria*, we discovered a remarkable degree of variation in male-specific abdominal coloration. In testing the expression patterns of the major pigment-producing enzymes, we found that phenotypes uniquely correlated with differences in the expression of *ebony*, a gene required for yellow-colored cuticle. Assays of *ebony’s* transcriptional control region indicated that a lightly pigmented strain harbored *cis*-regulatory mutations that caused correlated changes in its expression. Through a series of chimeric reporter constructs between light and dark strain alleles, we localized function-altering mutations to a conserved silencer that mediates a male-specific pattern of *ebony* repression. This suggests that the light allele was derived through the loss of this silencer’s activity. Furthermore, examination of the *ebony* gene of *D*. *serrata*, a close relative of *D*. *auraria* which secondarily lost male-specific pigmentation revealed the parallel loss of this silencer element. These results demonstrate how loss-of-function mutations in a silencer element resulted in increased gene expression. We propose that the mutational inactivation of silencer elements may represent a favored path to evolve gene expression, impacting morphological traits.

## Author Summary

One of the greatest challenges in understanding the relationship between genotype and phenotype is to discern how changes in DNA affect the normal functioning of genes. Mutations may generate a new function for a gene, yet it is frequently observed that they inactivate some aspect of a gene’s normal capacity. Investigations focused on understanding the developmental basis for the evolution of anatomical structures has found a prevalent role for mutations that alter developmental gene regulation. In animals, genes are transcriptionally activated in specific tissues during development by regulatory sequences distributed across their expansive non-protein coding regions. Regulatory elements known as silencers act to prevent genes from being expressed in certain tissues, providing a mechanism for precise control. Here, we show how a silencer that prevents expression of a pigment-producing enzyme in certain *Drosophila* species has repeatedly been subject to inactivating mutations that increased this gene’s expression. This example illustrates how such negative-acting regulatory sequences can represent a convenient target for increasing gene expression through the loss of a genetic element.

## Introduction

The role of repression in transcriptional regulation dates back to our initial glimpses of its molecular mechanisms [1]. While activating transcriptional control sequences, referred to as “enhancers”, contain binding sites for both activating and repressive transcription factors [2], some repressors act globally within gene loci to prevent the activation of multiple enhancers [3]. Such long-range inputs into gene regulation are contained within negative-acting sequences, often referred to as “silencers” [4]. Our current understanding of silencer function and evolution has lagged far behind that of the positive-acting enhancers. This is, in part, due to the difficulty associated with identifying negatively-acting elements in the expansive non-coding regions surrounding genes.

A growing number of studies have demonstrated how the alteration of gene regulation is critical to the evolution of morphology [5]. Hundreds of examples of gene regulatory sequence evolution have now been identified [6,7], and many of these have been shown to directly affect morphology [8–12]. In particular, many traits within *Drosophila* have provided a fruitful platform in which to connect phenotypic differences to changes in gene regulatory sequence [13–15]. These cases frequently require the characterization of complex regulatory regions that have multiple enhancer elements. However, published studies of gene regulatory evolution have generally focused on positively-acting enhancers, and have not addressed the role of negative-acting silencers in the evolution of gene expression, or their role in generating phenotypes.

The varied pigment patterns of the Drosophilid abdomen have provided fertile ground for advancing an understanding of gene regulatory evolution and its impact on phenotype [16–21]. The Drosophilid abdomen is divided into flexible segments protected by cuticular plates known as tergites that are secreted by an underlying epithelium. Patterned expression of melanin synthesis enzymes within this epithelium determines the ultimate cuticle pigmentation phenotype. In particular, coordinated expression of the genes *yellow*, *tan*, and *ebony* are broadly associated with pigment patterns across a wide range of species [8,17,19,21].

Previous work on the regulation of *ebony* in the abdominal epithelium has revealed a complex architecture of enhancers and silencers that govern its patterning [18]. *ebony* mRNA is specifically restricted from the posterior body segments of *Drosophila melanogaster* males to promote sexually dimorphic pigmentation. In more anterior body segments, *ebony* transcripts are limited to the anterior portion of each tergite that will produce a yellow color. A positively acting enhancer located 3.7 kb upstream of the transcription start site drives expression throughout the abdomen, a pattern that is ectopic relative to the endogenous pattern of *ebony* mRNA. This ectopic activity is restricted *in vivo* by two silencers. One silencer is located between 1.5 and 1.3 kb upstream of the promoter, and is required for the sexually dimorphic restriction of *ebony* transcripts from the male abdomen. A second silencer element residing within the first intron of *ebony* further restricts activity from the posterior edges of tergites. In a previous study, we demonstrated that both silencer activities were conserved to *D*. *prostipennis*, a species closely related to *D*. *melanogaster* [21]. The complex regulatory apparatus of the *ebony* locus stimulates the question of how silencers participate in the evolution of gene regulation.

Here, we explore the genetic basis of intraspecific variation in pigment patterns within the *montium* subgroup species *D*. *auraria* across Japan, and find that transcriptional silencers play a key role. Despite invariant patterns of *tan* and *yellow* expression, pigmentation patterns in this species correlated with *ebony* gene expression. Examination of the *D*. *auraria ebony* gene revealed that the upstream male-specific silencer element present in *D*. *melanogaster* is conserved, as it is located in a similar position relative to the *ebony* promoter. By localizing mutations in *cis* to *ebony*, we found that this conserved silencer was mutationally inactivated in a light *D*. *auraria* strain, an event that we found to be repeated in an additional *montium* subgroup species, *D*. *serrata*. The parallel inactivation of the same element suggests that the mutational loss of silencer elements may provide a simple and frequently traversed evolutionary path to increases in gene expression.

## Results

### Intraspecific variation of abdominal pigmentation within *D*. *auraria* populations from the islands of Japan

**Fig 1:**
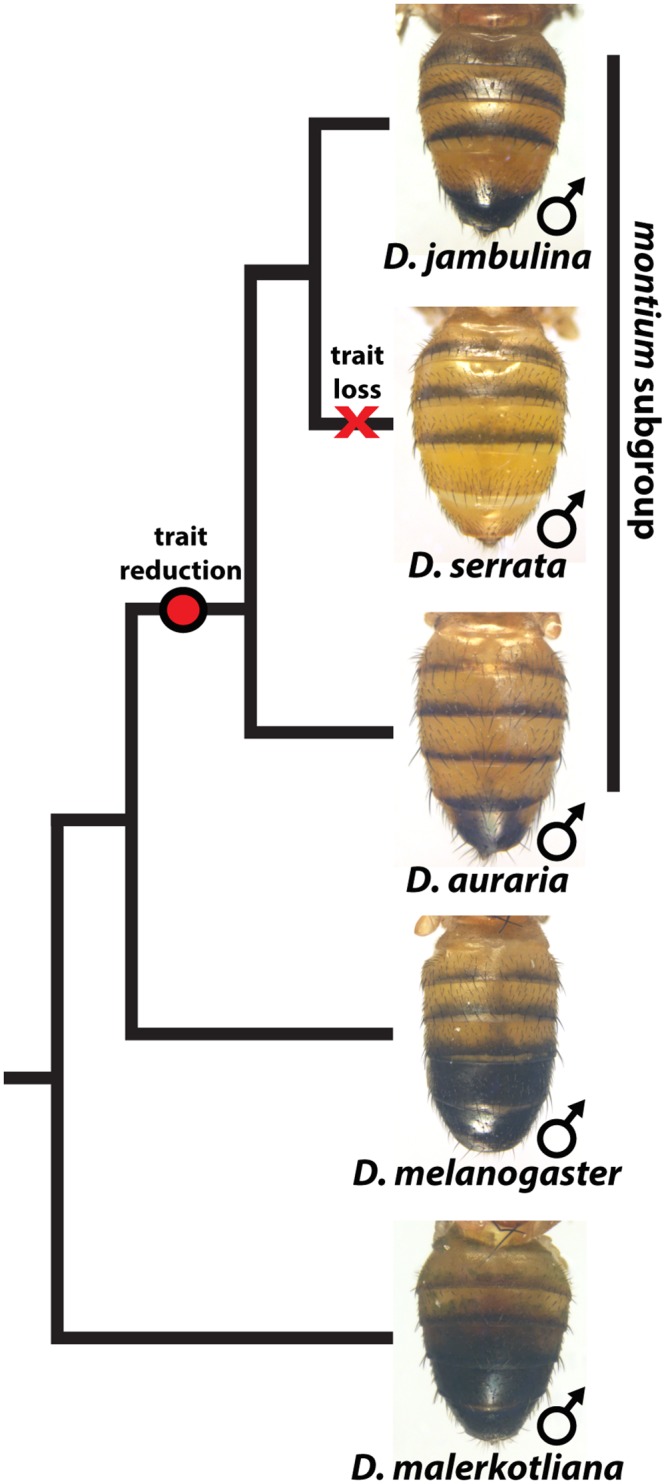
The loss and diversification of body pigmentation in males of the *montium* subgroup. Within the *montium* subgroup, the pigmentation phenotype of males has been reduced from two tergites (as exemplified by *D*. *melanogaster*) to one tergite (as in *D*. *auraria* and *D*. *jambulina*). *D*. *serrata* represents a case in which male-specific pigmentation has been lost.

**Fig 2:**
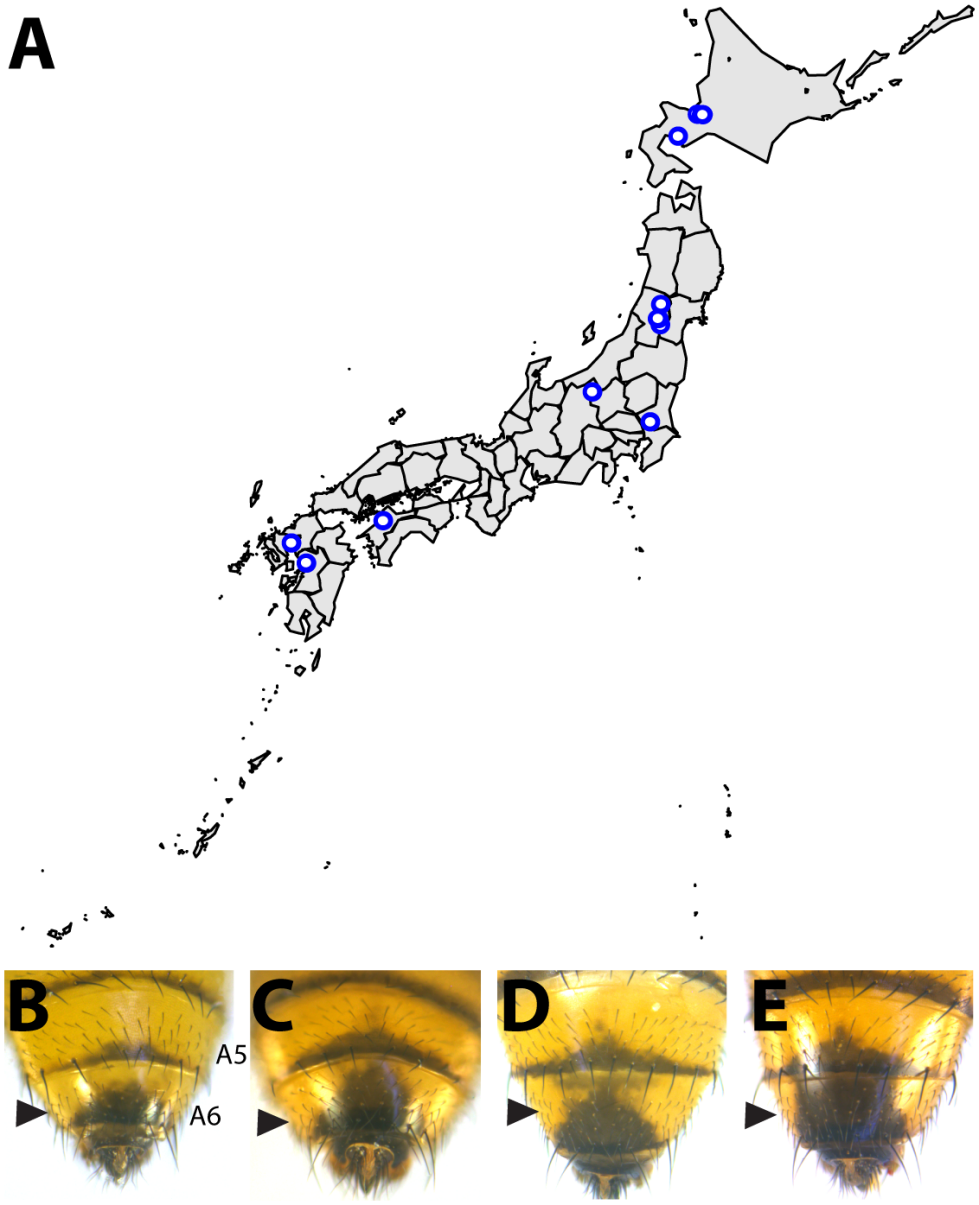
Intraspecific variation in abdominal pigmentation of *D*. *auraria* across Japan. (A) Map of the Japanese islands, depicting sites (blue dots) from which *D*. *auraria* strains in this study were collected. (B-E) The abdominal phenotypes observed in *D*. *auraria* range from light (B) to intermediate (C, D), to dark (E).

While the degree of female pigmentation is often highly variable within Drosophilidae species [22], variation in male coloration is relatively rare [20,21]. Our examination of *D*. *auraria* strains from Japan revealed an unexpected diversity of male-specific pigmentation patterns. In the *montium* subgroup, males exhibit reduced pigmentation limited to the single posterior-most tergite, as exemplified by *D*. *auraria* (Fig 1). Pigmentation in this group is also marked by several parallel losses of melanic color, such as the case of *D*. *serrata* (Fig 1) [16,21]. Among different *D*. *auraria* populations, the male phenotype continuously varied from dark individuals in which the entire A6 segment was pigmented to substantially lighter individuals in which dark pigmentation was limited to the posterior edge of the A6 tergite (Fig 2B–2E). The fully pigmented dark phenotype mirrors other closely related outgroups within the *montium* subgroup (Fig 1), supporting the inference that light pigmentation is the derived state within this species. We were next curious how this variation might align with environmental variation.

Many examples of clinal variability in insect pigmentation show trends of correlation between pigmentation and altitude or longitude [23]. In fact, within *D*. *auraria*, one such cline in female pigmentation was observed across Korea [24]. To determine whether male pigmentation varies clinally, we quantified the average pigmented area of A6 tergites for 29 isofemale lines of *D*. *auraria* across Japan (Fig 2A and Table 1). There was no significant correlation between this character and altitude, longitude, or latitude (S1 Fig). However, we noted a high degree of variation within each line (Table 1), suggesting that the light phenotype may be quite prevalent among the natural populations. Each isofemale line contains not only the genetic composition of the female that was caught, but also that of one or more males. As isofemale strains are cultured for multiple generations in the lab, this genetic variation would be expected to drift somewhat randomly. Because of this, we cannot be sure if the average pigmentation of a line is representative of the population from which it was derived. To look at the geographic prevalence of different phenotypes, we assessed the presence or absence of the dark or light phenotype within each line. We observed that lines in which the dark phenotype was present were collected from higher latitudes compared to lines in which this phenotype was absent (logistic regression: λ^2^_1_ = 7.09, p = 0.0078, S1F Fig). This data suggests that the dark phenotype may be maladaptive at lower latitudes. We next set out to elucidate the molecular mechanisms underlying the observed phenotypic variation within the *D*. *auraria* population.

**Table 1:** *D*. *auraria* strains surveyed for the geographic distribution of pigmentation phenotypes.

Strain	Latitude (N)	Longitude (E)	Altitude (m)	N individuals phenotyped	Light Phenotype Present	Intermediate Phenotype Present	Dark Phenotype Present	Percent of A6 Pigmented	STD DEV (%)	SEM (%)
A12	35.689506	139.6917	37	7			x	77.4%	7.63%	2.9%
A541	36.033333	140.066667	22.517	12	x			38.5%	9.58%	2.8%
KMM8	32.8031004	130.7078911	22.222	27	x	x		59.1%	9.95%	1.9%
MT14-1	33.774	132.798	64	5	x	x	x	55.9%	15.19%	6.8%
MT14-3	33.774	132.798	64	23		x	x	74.9%	10.56%	2.2%
MT14-7	33.774	132.798	64	3		x		78.0%	10.92%	6.3%
NGN-13	36.717635	138.4920422	1492.811	3	x	x		46.8%	7.02%	4.1%
NGN-27	36.717635	138.4920422	1492.811	2		x		49.6%	3.98%	2.8%
NGN11	36.717635	138.4920422	1492.811	5			x	69.7%	4.53%	2.0%
NPR13	43.0564977	141.496356	37.582	2		x		49.5%	13.76%	9.7%
NPR14	43.0564977	141.496356	37.582	8			x	79.3%	5.45%	1.9%
NPR15	43.0564977	141.496356	37.582	23	x	x	x	58.0%	14.54%	3.0%
SGA-1	33.263482	130.3008576	7.076	6	x	x		42.7%	5.33%	2.2%
SP11-11	43.066667	141.35	28.263	15		x	x	73.3%	8.58%	2.2%
SP11-12	43.066667	141.35	28.263	5			x	70.4%	8.02%	3.6%
SP11-14	43.066667	141.35	28.263	6			x	79.1%	11.38%	4.6%
SP11-15	43.066667	141.35	28.263	12	x	x	x	69.9%	11.85%	3.4%
SP96-2	43.066667	141.35	28.263	8		x	x	73.1%	16.62%	5.9%
SPP24	43.066667	141.35	28.263	6	x	x	x	48.4%	8.58%	3.5%
SPP26	43.066667	141.35	28.263	11	x	x	x	45.6%	13.61%	4.1%
SPP36	43.066667	141.35	28.263	9			x	71.7%	3.91%	1.3%
TUY20	42.565816	140.82375	88.109	17		x	x	71.2%	7.72%	1.9%
TUY21	42.565816	140.82375	88.109	17		x	x	61.2%	10.12%	2.5%
W220-nig	38.2554388	140.3396017	146.097	11		x	x	66.5%	5.50%	1.7%
YSG02	38.3809635	140.2759667	102.778	19		x	x	71.6%	11.40%	2.6%
YSG03	38.3809635	140.2759667	102.778	11	x	x		49.4%	6.84%	2.1%
YSG04	38.3809635	140.2759667	102.778	5	x	x	x	43.5%	15.69%	7.0%
YSG05	38.3809635	140.2759667	102.778	5	x	x	x	59.7%	14.83%	6.6%
YSG06	38.71565661	140.3526834	97.4555	14	x	x		39.3%	11.83%	3.2%

### Variation in abdominal pigmentation correlates with differences in *ebony* expression

Three genes, *yellow*, *tan* and *ebony*, are known to play a major role in the patterning of pigment through the enzymatic conversion dopamine derivatives [25–27]. The activity of Yellow is required for the production of dark, black pigment while Ebony converts dopamine intermediates to yellow-colored sclerotin. Tan promotes darker pigments by catalyzing the reciprocal reaction to Ebony. To date, these genes have been found to be expressed in a coordinated pattern in which *yellow* and *tan* are co-expressed [17], while *ebony* is expressed in a reciprocal pattern [18,28]. Currently, these genes encode the only enzymes in the pathway known to exhibit sharp expression patterns during abdominal development. Previously, it was shown that these correlated and mutually excluded spatial relationships of *yellow*, *tan*, and *ebony* expression are preserved during evolutionary shifts in pigment pattern [8,17,21].

**Fig 3:**
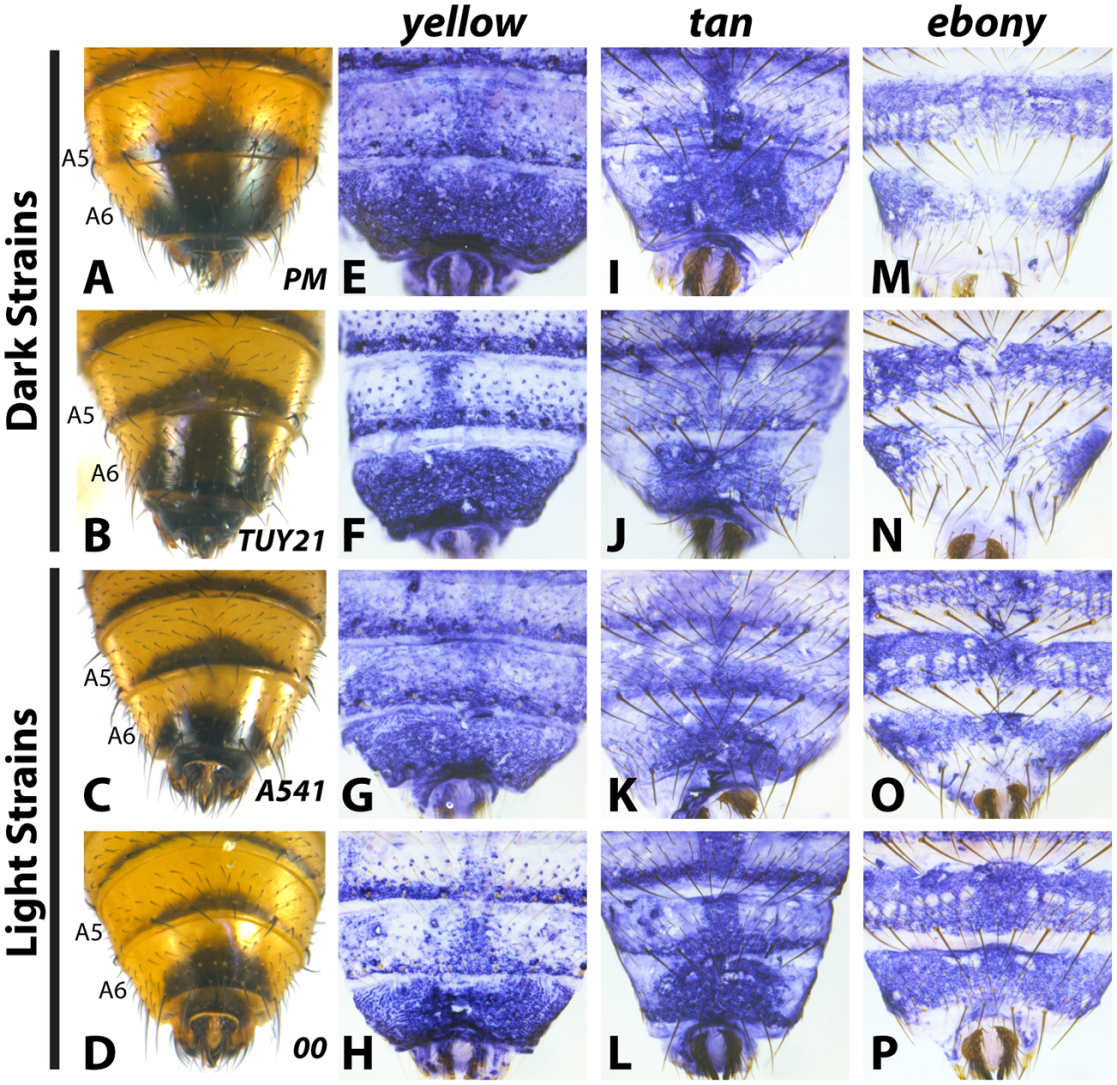
Variable abdominal pigmentation phenotypes within *D*. *auraria* uniquely correlate with *ebony* expression. (A-D) Abdominal phenotypes of dark (A, B), and light strains (C, D). (E-P) *in situ* hybridizations to visualize the accumulation of *yellow*, *tan*, or *ebony* mRNA in the PM strain (E, I, M), TUY21 (F, J, N), A541 (G, K, O), and 00 (H, L, P). While *yellow* and *tan* are similarly expressed among light and dark strains, *ebony* is absent in the midline of dark strains (M, N), and expressed evenly throughout the A6 tergite of light strains (O, P). Further information on strains is available in Table 1 and S1 Table.

To evaluate how the pigmentation pathway had been modified within *D*. *auraria*, we compared the expression of *yellow*, *tan* and *ebony* in strains for which males consistently had dark or light pigmentation. Surprisingly, both *yellow* and *tan* expression was indistinguishable among strains, despite striking differences in phenotype. *yellow* was expressed throughout the A6 segment and in repeated patterns along the posterior edges of each tergite (Fig 3E–3H and S2). Regardless of phenotype, this pattern did not differ between light and dark strains. *in situ* hybridizations localizing *tan* revealed expression restricted to the stripe pattern along the posterior edges of tergites, as well as a pattern along the midline of the A6 tergite that strongly foreshadows the pigmentation phenotype of dark strains (Fig 3I–3L and S3). However, this midline pattern of *tan* also appeared in lighter strains that lack this pigmentation phenotype. Our expression data with *tan* and *yellow* provide an example in which these two genes exhibit non-overlapping patterns of expression. Further, this represents a rare example in which *yellow* and *tan* expression was found to poorly correlate with pigmentation phenotype.

In contrast to *yellow* and *tan*, *ebony* expression correlated well with variation in male tergite pigmentation. Dark strains of *D*. *auraria* express *ebony* throughout the abdomen, save for the A6 tergite, in which only the lateral edges broadly accumulate transcript (Fig 3M and 3N). The medial region of A6 lacks *ebony* transcript, correlating with the dark pigmentation that forms in this region. For light strains, we observed that *ebony* expression was expanded into the dorsal portion of the A6 tergite in a pattern that correlates with this derived phenotype (Fig 3O and 3P). In variable strains that have mixtures of phenotypes, we observe both light and dark *ebony* expression phenotypes (S4 Fig). These results are consistent with a role of *ebony* in patterning the variable pigmentation phenotypes of *D*. *auraria*. We next set out to characterize the *D*. *auraria ebony* regulatory region to determine what role it may play in the diversity of *D*. *auraria* male pigmentation phenotypes.

### A conserved silencer element dictates the absence of *ebony* expression in male abdominal segments

**Fig 4:**
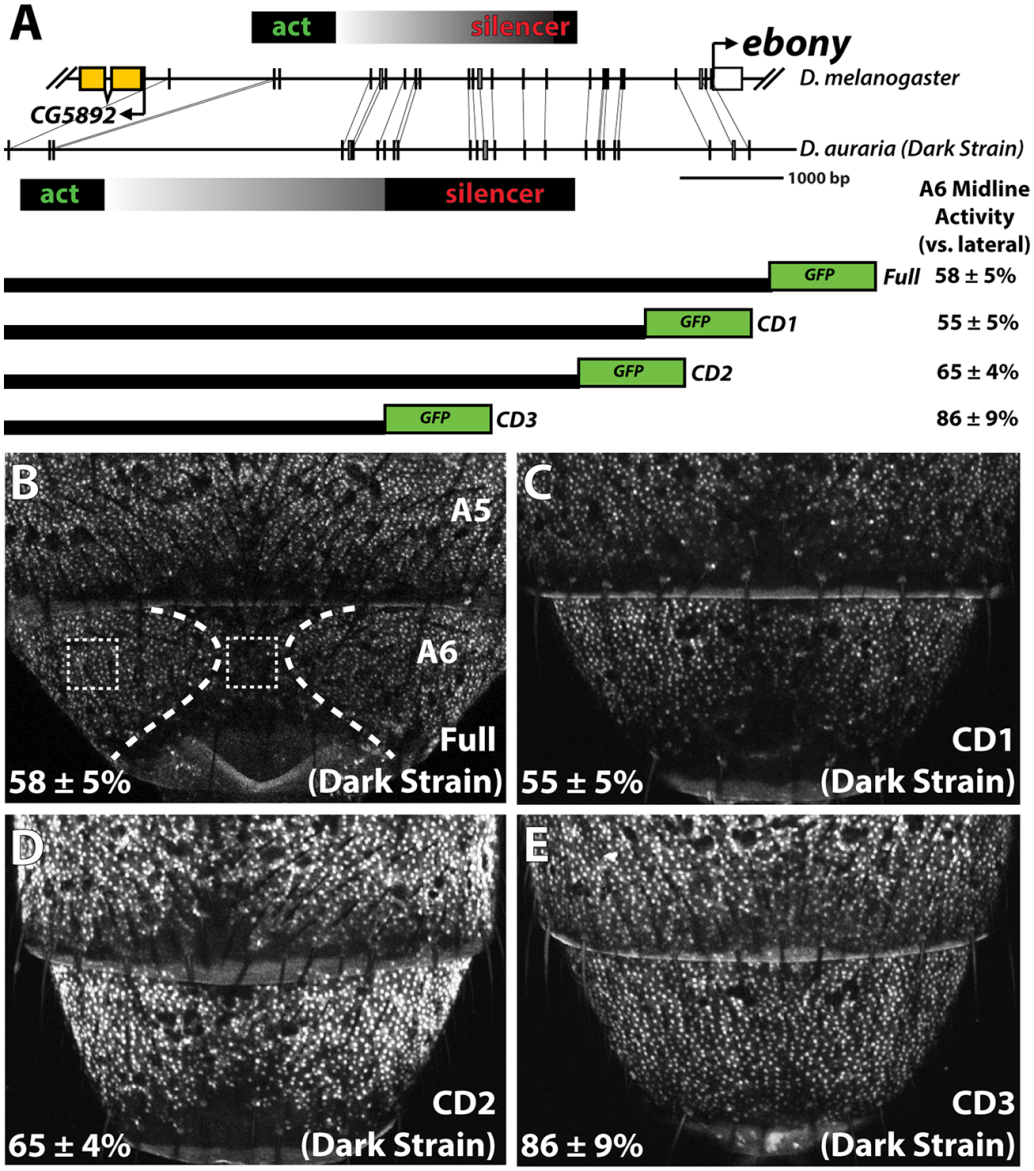
Localization of a conserved silencer in the *D*. *auraria* upstream regulatory region. (A) Schematic depicting the upstream regulatory region of *ebony* from a dark *D*. *auraria* strain aligned to the orthologous region of *D*. *melanogaster*. Gray boxes connecting *D*. *auraria* and *D*. *melanogaster* sequences indicate the relative position of perfectly conserved stretches. Positions of the minimal abdominal enhancer and silencer are shown above the *D*. *melanogaster* sequence are based on [18]. Position of the *D*. *auraria* silencer element is based upon the truncation constructs shown below the conservation plot. Regions experimentally determined to be required for silencer activity are listed in black, while the region between the enhancer and silencer are shown in a gray gradient, as they may contribute to silencing activity. “A6 midline activity” was measured for each truncation construct, in which the fluorescent intensity of the midline was expressed as the percentage of the lateral A6 intensity ± S.E.M. (B) Activity of the dark (“PM”) strain full *ebony* upstream regulatory region, which recapitulates the midline repression of *ebony* observed by *in situ* hybridization (dotted lines). Dashed boxes indicate representative midline and lateral patches used to quantify midline activity. (C-D) The CD1 truncation construct (C), and the CD2 construct (D) both show midline repression similar to that observed in the full *ebony* upstream region. (E) The CD3 truncation construct shows uniform expression of GFP across the A6 body segment, reflecting the elimination of sequences required for repression.

The pattern of *ebony* expression in *D*. *melanogaster* is controlled by at least three interacting *cis*-regulatory elements [18]. These regulatory activities are revealed in transgenic reporter assays when, in isolation, the distal activating enhancer drives strong expression in both endogenous and ectopic abdomen regions [18,29]. The ectopic activity of this enhancer is counterbalanced by a promoter proximal silencer element that prevents expression in the pigmented posterior segments of males (depicted in Fig 4A). Although this upstream enhancer and silencer architecture is conserved within the oriental lineage [21], the full extent of its conservation remains unknown. Our finding that *ebony* transcript is reduced in the pigmented A6 body segment of *D*. *auraria* males raised the possibility that a conserved mechanism governs the dimorphic expression of this gene.

We cloned the entire upstream region of *ebony* from a dark strain of *D*. *auraria* (“PM”, Fig 3A) into a green fluorescent protein (GFP) reporter vector (Fig 4A and S5 Fig). This reporter construct, which included the region orthologous to the abdominal enhancer and the male-specific silencing element was tested in transgenic *D*. *melanogaster*. We found that the reporter transgene’s expression precisely recapitulated the lateral pattern of *ebony* expression observed in the A6 tergites of dark *D*. *auraria* males (compare Fig 4B to Fig 3M). To determine if an orthologous male-specific silencer sculpts this pattern in *D*. *auraria*, we tested a series of truncations that would remove this potential silencer, leaving behind only sequences orthologous to the activating enhancer (Fig 4A). The first truncation had no effect on the lateral pattern of expression (Fig 4C). A larger truncation, CD2 resulted in marginally reduced, but visible repression along the dorsal midline (Fig 4D). The third truncation, which removed all sequences orthologous to the *D*. *melanogaster* male-specific silencer, resulted in a nearly complete elimination of midline repression (Fig 4E). These results establish the ancestrally conserved regulatory architecture at *ebony*, in which a conserved silencer collaborates with a pan-abdomen activating enhancer to sculpt out zones of contrast in expression.

### *cis*-regulatory changes at *ebony* inactivated the male-specific silencer in a light *D*. *auraria* strain

**Fig 5:**
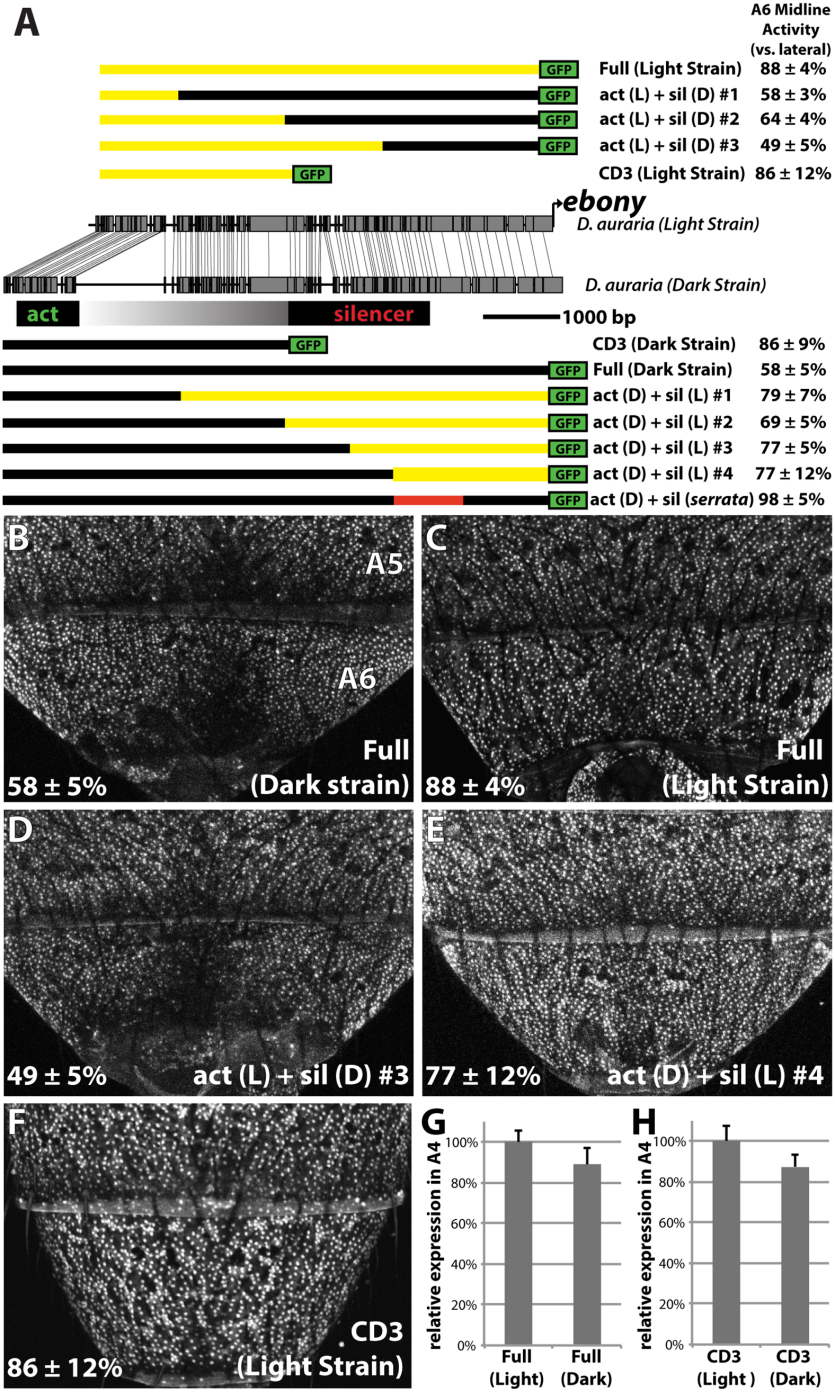
Changes in *cis* to the upstream silencer element of *ebony* are responsible for a difference in *ebony* expression between strains. (A) Comparison of the DNA sequence of the light and dark strains of *ebony*, as in Fig 4A. Schematics of GFP reporter constructs that pinpoint changes to the silencer of *ebony* are shown above and below the conservation plot. Chimeras appearing below the dark strain are drawn relative to the dark strain due to the presence of a large insertion in this strain. Similarly, chimeras above the light strain are drawn relative to the light strain due to the lack of this repetitive region. A6 midline activity was calculated using the same method described in Fig 4. The act (D) + sil (*serrata*) construct in (A) contains the 1kb silencer region of *D*. *serrata* placed in the context of the dark *D*. *auraria* strain *ebony* upstream regulatory region (Fig 6C). (B) Activity of the dark (“PM”) strain *ebony* upstream regulatory region, containing both activating enhancer and silencer region. Expression is specifically reduced in the dorsal midline. (C) Activity of the light (“00”) strain *ebony* upstream regulatory region, orthologous to that used for the dark strain. This reporter construct shows a marked increase in midline activity, recapitulating the *ebony* expression phenotype of this strain. (D) Chimeric reporter containing the light strain activating enhancer fused to the dark strain upstream silencer element. This reporter construct shows midline repression similar to the dark strain. (E) Chimeric reporter containing the dark strain activating enhancer fused to the light strain upstream silencer region, which recapitulates the light strain activity. (F) The CD3 truncation of the light strain reporter drives expression throughout the A6 segment, similar to the same truncation of the dark strain. (G) Graph comparing the relative activity of the Dark and Light strain Full *ebony* upstream reporter activity in the A4 body segment, a region unaffected by silencer activity. (H) Graph comparing the relative activity of the Dark and Light strain CD3 truncation constructs in the A4 body segment.

The presence of a conserved silencer in the *ebony* upstream region that controls the midline repression of *ebony* in the A6 segment raised the intriguing possibility that this silencer may have been inactivated in light strains. To compare the function of *ebony* regulatory sequences between light and dark strains, we cloned the orthologous upstream region of *ebony* from a light strain (“00”, Fig 3D) into our transgenic reporter system. The light and dark strains differed by several sequence polymorphisms, including a large insertion/deletion of repetitive sequence near the activating enhancer that is absent in the light strain sequence (Fig 5A). We inserted a transgenic construct containing the light strain’s *ebony* upstream region into the same genomic landing site that was used for our tests of the dark strain sequence. Although the light and dark strains differed slightly in activity in the more anterior A4 body segment (Fig 5G), the light strain’s regulatory region recapitulated its expanded expression throughout the A6 tergite relative to the dark strain (Fig 5C). From this, we conclude that the observed differences in *ebony* expression between light and dark *D*. *auraria* strains are due to *cis*-regulatory mutations.

Next, we were curious whether the mutations in the *ebony* upstream region were localized to the known activating or silencing CREs, or outside of these defined activities. To directly compare the activities of the abdominal enhancer region between light and dark strains, we cloned the CD3 truncation from the light strain construct (Fig 5A). As expected, this region drove expression throughout the A6 tergite (Fig 5F). The light and dark CD3 constructs subtly differed (by 13%) in the intensity of expression driven in the A4 body segment (Fig 5H). These data indicate that some differences may exist in the abdominal enhancer between the light and dark strains.

To localize the mutations responsible for differences in A6 midline repression between light and dark strains, we generated a series of chimeric reporter constructs in which a segment of the dark or light strain *ebony* upstream region was replaced with the analogous segment from the other strain (Fig 5A). Among these chimeric reporters, the phenotype of expression was dictated by which allele was present at the promoter-proximal silencer element. This is exemplified by the phenotypes of two constructs (Fig 5A), in which a 2.2 kb promoter proximal fragment containing the silencer could switch activity from the dark expression phenotype to the light phenotype or *vice versa* (Fig 5D and 5E). Chimeric reporters that contained larger segments surrounding the silencer element similarly displayed the coinciding phenotype of the allele present at the silencer element (Fig 5A and S6 Fig). However the degree of male repression quantitatively differed in subtle, but repeatable ways. Specifically, chimeras whose breakpoints were located at the center of the upstream region displayed more intermediate repression phenotypes (“act(L) + sil (D) #2”, “act(D) + sil(L) #2 Fig 5A and S6 Fig). This suggests the presence of mutations in the light strain sequence that can enhance repression but are context dependent. Overall, these observations establish that the increase in *ebony* expression in the light strain occurred primarily through mutations affecting the *ebony* male-specific silencer element.

### The parallel loss of the *ebony* male-specific silencer function accompanied the loss of pigmentation in *D*. *serrata*

**Fig 6:**
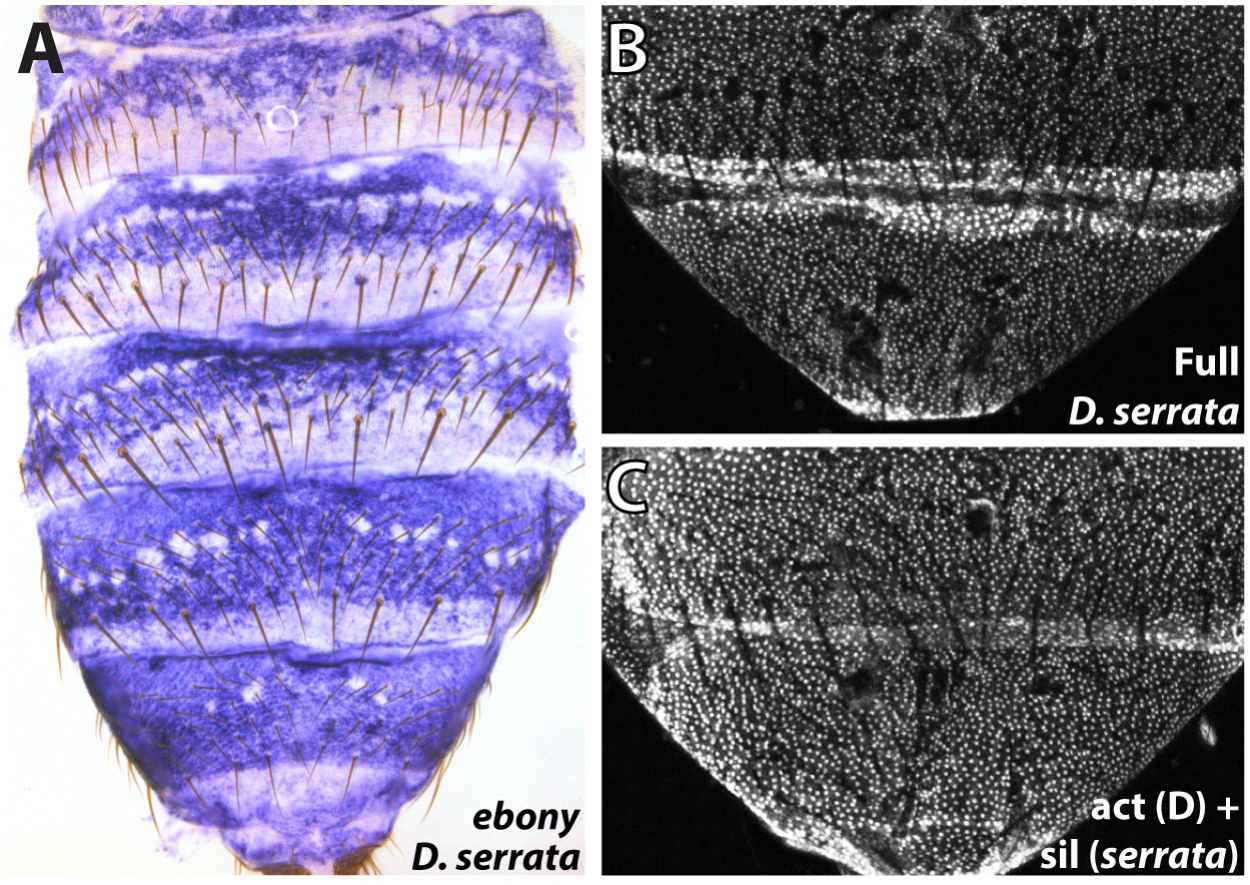
Parallel inactivation of the *ebony* upstream silencer in *D*. *serrata*. (A) Expression of *ebony* in the abdomen of a *D*. *serrata* male (UCSD “03” strain). (B) The entire upstream region of *D*. *serrata ebony* fused into a GFP reporter construct recapitulates the broad activation of *ebony* throughout the male posterior abdomen. (C) Chimeric reporter in which a 1 kb segment containing the *D*. *serrata* upstream silencer region was placed into the *D*. *auraria* dark strain reporter construct. This reporter lacks midline repression, indicating that the *D*. *serrata* upstream silencer was inactivated.

The inactivation of existing functional elements is often a favored mechanism during evolutionary change [30]. We were therefore curious whether a similar evolutionary path marked parallel alterations in pigmentation. *Drosophila serrata* is a *montium* subgroup species that has secondarily lost male-specific pigmentation (Fig 1). Examination of *ebony* expression in *D*. *serrata* males revealed that four independent lines exhibited broad expression throughout the posterior segments (Fig 6A and S7 Fig). To test whether this alteration in *ebony* expression in *D*. *serrata* was due to *cis*-regulatory mutations in the gene, we cloned its orthologous upstream region including the activating enhancer and promoter-proximal silencer into our transgenic reporter system (S5 Fig). Consistent with a *cis*-regulatory basis for this expression phenotype, the *ebony* upstream reporter recapitulated the endogenous *D*. *serrata ebony* expression pattern (Fig 6B). To test whether this was indeed due to a mechanism similar to that observed in *D*. *auraria*, we replaced a 1 kb segment containing the male-specific silencer of the *D*. *auraria* dark strain with orthologous sequences from *D*. *serrata* (Fig 5A, “act(D) + sil(*serrata*)”). This chimeric construct drove expression throughout the A6 body segment similar to that of the full *D*. *serrata ebony* sequence (compare Fig 6C to 6B), confirming the parallel inactivation of this silencer element.

## Discussion

**Fig 7:**
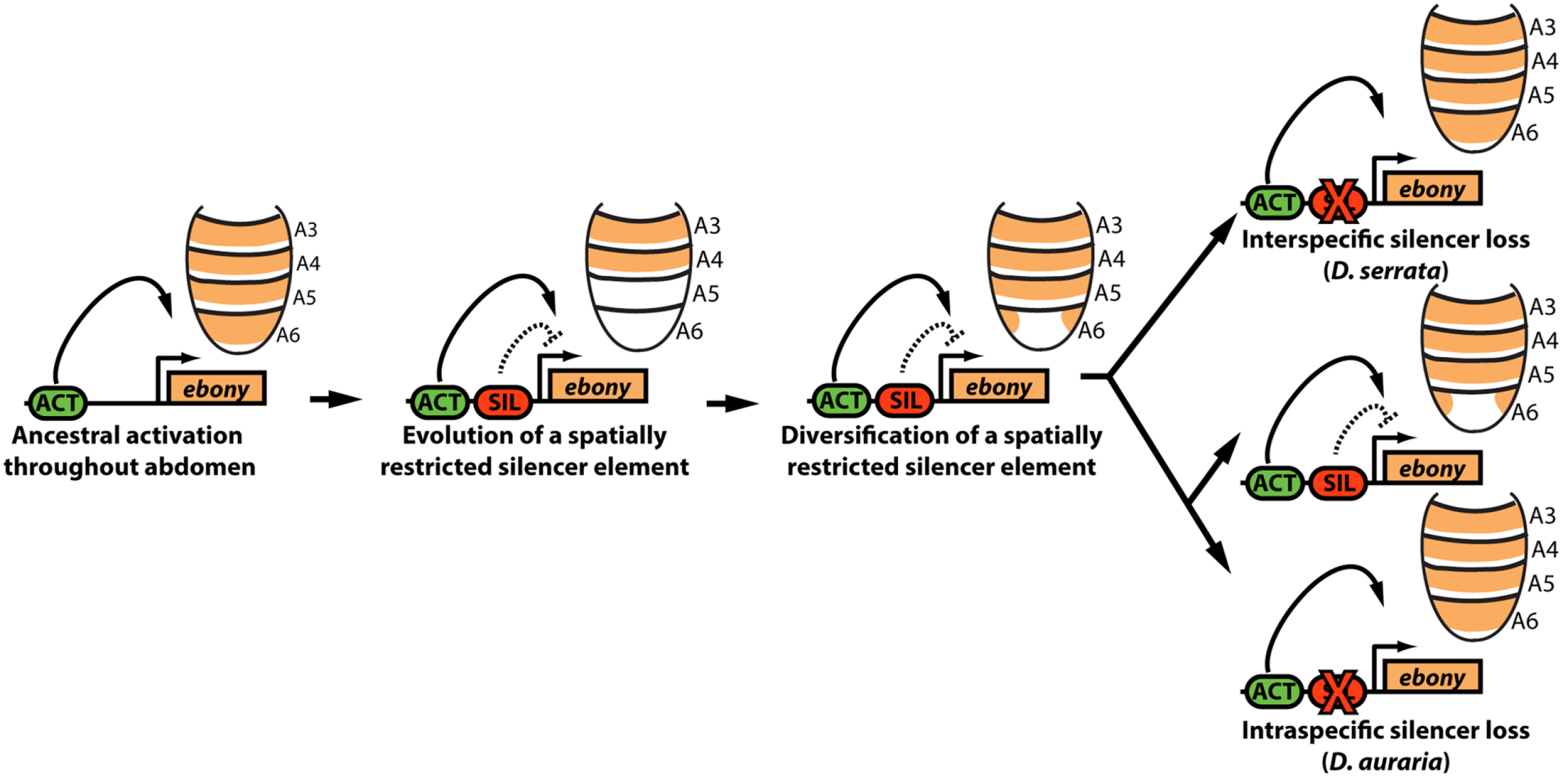
Model for the parallel loss of the *ebony* upstream silencer element. Within the ancestral *ebony* gene, a silencer element evolved that adopted a repressive role in in the male posterior abdomen. Within the *montium* subgroup, this silencer’s activity was modified through changes in *cis* that altered the silencer’s spatial domain of activity. Within *D*. *serrata*, this silencer was inactivated, leading to increased *ebony* expression. In contrast, this silencer was maintained in the species *D*. *auraria*, in which intraspecific variation exists for an allele that inactivated this element.

Here, we have shown how a transcriptional silencer that participates in the sexually dimorphic patterning of gene expression has experienced repeated inactivation events that increased its target gene’s expression (Fig 7). Despite highly varied male pigmentation phenotypes in *D*. *auraria*, two genes that often correlate with pigment pattern (*yellow* and *tan*) were expressed similarly among light and dark strains. In contrast, *ebony* expression uniquely correlated with pigmentation, and we showed that the correlation between expression and pigmentation phenotypes were due to *cis*-regulatory mutations within *ebony*. Mapping the *ebony* regulatory region of *D*. *auraria* established the conservation of a silencer element with male-specific activity that carves out sexually dimorphic expression from a ubiquitously activating enhancer. Within this conserved silencer, we localized functional changes responsible for allelic differences in expression. Moreover, we showed that the secondary loss of male-specific pigmentation in *D*. *serrata* occurred through the parallel inactivation of this upstream silencer element of *ebony* (Fig 7). These findings highlight the under-appreciated role of silencers in the evolution of gene expression and morphology. We briefly discuss the implications of these results within a growing body of knowledge regarding the evolution of gene regulation.

### Potential causes for variable pigmentation in *D*. *auraria*

While many differences in female-specific or body-wide pigmentation have been described among Drosophilids [20,22,31,32], examples of variation in male-specific pigmentation are comparably lacking. Intriguingly, a clear cline exists for *D*. *auraria* females in Korea, and yet the pigmentation phenotypes of males in this population were reported to be invariant [24]. From our survey across Japan, a distinct trend emerged in which females were relatively invariant, while males exhibited a remarkable degree of variability. Several adaptive mechanisms have been invoked to explain phenotypic variation in pigmentation (e.g. thermoregulation [33], desiccation resistance [34,35], UV resistance [36]). However, examples that contradict these trends highlight how pigmentation does not confer a “one size fits all” universal benefit [37,38]. Our tests for geographical correlations failed to support a traditional cline, though this may have been hampered by the small number of lines tested and the high degree of phenotypic variation within each line (Table 1). Indeed, when we controlled for intra-line variation by characterizing the range of phenotypes contained within, we found that lines bearing the dark phenotype occurred at higher latitudes than those lacking this phenotype (S1F Fig). This result follows the general trend of latitudinal clines for pigmentation [39,40], and suggests that the dark phenotype may be maladaptive at lower latitudes. Given the high degree of variation present within the population, a further examination of wild-caught males, possibly taking into account other factors such as seasonal variation or habitat structure may reveal forces that have shaped the phenotypic variation observed within this species.

### The uncoordinated pigmentation gene network of *D*. *auraria*

The coordinated evolution of pigmentation enzyme expression has become the rule, rather than the exception with *Drosophila* coloration phenotypes [8,17,21,28,41]. Across the abdomen, expression of *yellow* and *tan* are highly correlated [17,21], while *ebony* is typically anti-correlated with these two [18,21]. Our data on the variable pigmentation of *D*. *auraria* provides an exception to this rule in which both *tan* and *yellow* are expressed in the A6 tergite, regardless of the pigmentation phenotype (Fig 3). Indeed, these two genes show consistent discord in their expression patterns, as *yellow* is expressed throughout A6 in contrast to the highly patterned expression of *tan* in the dorsal portion of the tergite. An additional layer of uncoordinated expression is introduced by *ebony*, which is deployed in the same cells as *yellow* and *tan* in strains that display the light phenotype. The lack of correlation of *tan* and *yellow* with pigmentation (Fig 3), and the inability of *ebony* mis-expression to erase all dark pigmentation in *D*. *melanogaster* in isolation [28] suggests that additional genes likely contribute to the variation we have observed in *D*. *auraria*.

Previously, we showed how a recent interspecific expansion of pigmentation involved coordinated changes in *yellow*, *tan*, and *ebony* [21]. In this example, the *yellow* gene was shown to have evolved an expanded pattern in *cis*, independent of *tan* and *ebony*, whose expression differences were due to changes in one or more upstream factors. Considering these results in light of our current findings, the structural genes of the *Drosophila* pigmentation gene network appear to quite readily evolve new domains of expression independent of one another.

### Loss, gain, and bias in the evolution of gene regulatory elements

An emerging theme from endeavors to connect genotype to phenotype is a bias towards certain molecular paths of evolution [6,42]. An ever-growing body of work has established that changes in gene regulation represent a commonly traversed path during the evolution of morphological differences [5,7]. It is thought that the specific genes that contribute to phenotypic variation may also be biased, due to their positions within networks [7]. This trend extends to the *Drosophila* pigmentation system, as *yellow*, *tan*, and *ebony* have been repeatedly implicated in cases of phenotypic evolution [8,16–19,21,31,43,44]. One common source of genetic bias is the tendency for loss rather than gain of genetic elements [6,30,42,45]. This is thought to be due mainly to mutational target size, as there are many more possible mutations that would inactivate a genetic element than those which would build a new one [42]. Consequently, if a phenotype can be achieved through inactivation, there will undoubtedly be many more possible degenerative routes than constructive ones. The present work highlights how the trend of loss extends to negative acting gene regulatory elements whose inactivation will increase gene expression.

Several examples of morphological evolution have been linked to the loss of transcriptional activating enhancer elements [9,13,17,43]. A striking example is that of the *shavenbaby* gene, in which multiple enhancers that contribute to its larval denticle patterning function were inactivated [13]. Despite the existence of many other loci that could contribute to this trait, some of these exact same elements were inactivated in an independent case of trichome loss, suggesting bias in the genetic path of evolution of this particular trait [46]. Because gene regulatory regions are subdivided into modular subunits that act relatively independently, the loss of an enhancer’s activity should be minimally pleiotropic, affecting only one or a small number of tissues. Similarly, the pleiotropic consequences of inactivating a single silencer element are predicted to be minimal, as effects will be limited to tissues where the silencer is actively suppressing an enhancer’s activity.

While the existence of long-range repressors in metazoan regulatory architecture has been appreciated for nearly three decades [4,47], their identification and characterization has lagged behind that of the enhancer elements that activate transcription. This disparity is almost certainly due to the way regulatory elements are experimentally characterized by fusing small (generally less than 10kb) overlapping fragments of potential regulatory DNA to reporter genes, and monitoring activity in the tissue of interest [29]. However, such tests will be at a disadvantage to uncover more complex relationships between elements, including insulators [48,49], promoter-tethering elements [50,51], and silencers [4,47]. As such, it is almost a certainty that many silencers have gone unnoticed within well-characterized regulatory regions. For example, ectopic activity is frequently observed when enhancers are trimmed down to minimal elements [52,53]. In the *ebony* regulatory region, ectopic activity of our reporter transgenes was conspicuous, given the large size of the adult abdominal epithelium and the sharply contrasting patterns of expression that we observed *in vivo* [18]. This stimulated a more comprehensive search of the locus for negative-acting elements.

The complexity of regulatory architecture at *ebony* lends itself well to disentangling the interplay of gain and loss of regulatory inputs during evolution. For example, our previous study of intraspecific variation at the *ebony* gene revealed a gain-of-function mutation that caused reduced expression in *D*. *melanogaster* [18]. Of five function-altering substitutions that reduced expression in a high altitude population, we observed that the largest effect substitution paradoxically resided outside of the activating enhancer for the abdomen. This contradiction was reconciled by an experiment in which the altered residue was deleted, leading to a marked recovery of enhancer activity. As we obtain a more detailed understanding of the molecular events underlying gene regulatory evolution, the distinction between loss and gain of function at the molecular versus genetic levels will become more frequently resolved. This is a crucial step in interpreting and predicting how regulatory variants influence phenotype.

## Methods

### Fly lines and husbandry

Fly stocks were maintained on standard media at room temperature. Isofemale lines of *D*. *auraria* were collected in hanging traps and GPS coordinates of the collection site were recorded. See Table 1 and S1 Table for a list of species and strains used in this study.

### Quantification of strain pigmentation

Strain pigmentation phenotypes were measured by collecting flies without CO_2_ anesthetization on the day of eclosion and aging 5 days at 25°C to normalize cuticular tanning. We then anesthetized aged flies, mounted the adult abdomens to double-sided sticky tape on slides, and imaged the abdomen using standard settings on a Leica M205 microscope. Images were quantified using the ImageJ program [54] to measure the area of the tergite that was pigmented, divided by the total area of the tergite, yielding a percent pigmentation score. The relationship between strain pigmentation and latitude, longitude, and altitude was analyzed by nominal logistic regression.

### *in situ* hybridizations

*in situ* hybridization was performed as previously described [17]. Templates for probes were cloned into the pGEM vector, and PCR amplified to contain an Sp6 or T7 promoter present in the pGEM multiple cloning site (see S2 Table for probe primers used in this study). *in vitro* transcription of probes was performed using a 10X Dig labeling mix (Roche Diagnostics) in combination with T7 or Sp6 RNA polymerase (Promega). Pupal samples were aged to differing extents for each probe (75–90 hours after pupal formation (hAPF) for *yellow*, 85–95 hAPF for *tan*, and at eclosion for *ebony*), dissected in cold PBS, and fixed in 4% paraformaldehyde (E.M.S. Scientific). All *in situ* hybridizations were performed using an Insitu Pro VSI robot (Intavis Bioanalytical Instruments).

### Transgenic reporter constructs

Fragments of the *ebony* gene were PCR amplified from genomic DNA using the primers presented in S3 Table. The light and dark strain constructs were amplified from the “00” and “PM” stocks (Fig 3 and S1 Table), while the *D*. *serrata* reporter was cloned from the UCSD “03” strain (S1 Table). Chimeric constructs were generated by overlap extension PCR to fuse light and dark strain non-coding regions together. PCR products were cloned via appended restriction sites into the S3aG vector [15], which contains a minimal promoter driving enhanced nuclear GFP, flanked by gypsy and Sfb insulators. The S3aG vector contains an attB site for site-specific integration into the genome [55]. Transgene constructs were inserted into the 51D landing site on the second chromosome [55] by Rainbow Transgenics. Multiple independent lines were analyzed for each construct.

Homozygous transformants were aged for 8–9 hours post-eclosion to maximize the signal to noise ratio of abdominal expression, as weak expression occurs in the abdomen during pupal development. Abdomens were mounted on slides in halocarbon oil, and imaged on an Olympus Fluoview 1000 confocal microscope using standardized non-saturated settings. Multiple images per line were acquired, and the degree of A6 midline expression was quantified by measuring the average intensity of a 50x50 pixel square at three positions along the A6 tergite (left, right, middle). The intensity value of the midline was divided by the average of the left and right side measurements.

## Supporting Information

S1 Fig Intraspecific variation in *D*. *auraria* pigmentation does not follow a traditional geographical cline. (A-C) Correlation of A6 pigmentation, expressed as the percent of the tergite pigmented, with latitude (A), longitude (B), and altitude (C). (D-F) Analysis latitude, taking into account variation within lines. Isofemale lines were scored based on whether the light (D), intermediate (E), or dark (F) phenotype was absent or present, and the average latitude of these lines were calculated. For the dark phenotype (Panel F), a significant difference in average latitude was detected (* logistical regression: λ^2^_1_ = 7.09, p = 0.0078).
(TIF)

S2 Fig *yellow* is expressed similarly in lines that contain the light or dark phenotype. *in situ* hybridization with a probe to *yellow* reveals similar patterns of *yellow* mRNA accumulation between strains containing light (and intermediate) phenotypes compared to those containing dark (and intermediate) phenotypes.
(TIF)

S3 Fig *tan* is expressed similarly in lines that exhibit the light or dark phenotype. *in situ* hybridization with a *tan* probe reveals a similar pattern of mRNA accumulation among strains that contain either the light (and intermediate) or dark (and intermediate) abdominal pigmentation phenotypes.
(TIF)

S4 Fig Variable *ebony* expression in lines that contain a mixture of light and dark pigmentation phenotypes. *in situ* hybridization with an *ebony* probe reveals lines containing a mixture of *ebony* expression phenotypes that either match the dark strain phenotype (i.e. reduced midline expression), or the light strain phenotype (uniform expression throughout the anterior portion of the A6 tergite).
(TIF)

S5 Fig Isolation of the *ebony* upstream region from *montium* subgroup species. Lines connecting sequences represent conserved sequence blocks of 12 bp or more shared between the species. The difference in length between dark and light *auraria* strains is caused by a repetitive sequence present in the dark strain sequence.
(TIF)

S6 Fig Chimeric constructs localize causative changes to the conserved promoter-proximal silencer region. Transgenic reporter lines were imaged 8–10 hours post-eclosion. A6 midline activity ± S.E.M. is shown in the lower-right corner.
(TIF)

S7 Fig The expansion of *ebony* expression is fixed among males in *D*. *serrata* strains. (top) Male phenotypes of *D*. *serrata* strains from the UCSD stock center. (bottom) *in situ* hybridization to *ebony* reveals gene expression throughout the posterior body segment that has lost pigmentation. Additionally, the extent of *ebony* mRNA in the posterior portion of each tergite matches pigment stripe phenotype for each strain.
(TIF)

S1 Table Additional strains used in this study. (DOCX)

S2 Table Primers for *in situ* hybridization probes. (DOCX)

S3 Table Primers for constructing reporter constructs, including chimeric reporters. Lowercase letters represent appended restriction sites for Asc I (Primer F) and Sbf I (Primer R). Primers for chimeras list the forward and reverse primers used in combination with the *D*. *auraria ebony* upstream primers (Primer-F and Primer-R) to generate two or more fragments that were fused together using overlap extension PCR.
(DOCX)

## References

[1] JACOB F, MONOD J (1961) Genetic regulatory mechanisms in the synthesis of proteins. J Mol Biol 3: 318–356. http://www.ncbi.nlm.nih.gov/pubmed/13718526. Accessed 2 September 2014. 13718526 10.1016/s0022-2836(61)80072-7

[2] Small S, Levine M (1991) The initiation of pair-rule stripes in the Drosophila blastoderm. Curr Opin Genet Dev 1: 255–260. http://www.ncbi.nlm.nih.gov/pubmed/1822273. Accessed 18 September 2014. 1822273 10.1016/s0959-437x(05)80079-6

[3] Gray S, Levine M (1996) Transcriptional repression in development. Curr Opin Cell Biol 8: 358–364. http://www.ncbi.nlm.nih.gov/pubmed/8743887. Accessed 18 September 2014. 8743887 10.1016/s0955-0674(96)80010-x

[4] Brand AH, Breeden L, Abraham J, Sternglanz R, Nasmyth K (1985) Characterization of a “silencer” in yeast: a DNA sequence with properties opposite to those of a transcriptional enhancer. Cell 41: 41–48. http://www.ncbi.nlm.nih.gov/pubmed/3888409. Accessed 18 September 2014. 3888409 10.1016/0092-8674(85)90059-5

[5] Carroll SB (2008) Evo-devo and an expanding evolutionary synthesis: a genetic theory of morphological evolution. Cell 134: 25–36. http://www.ncbi.nlm.nih.gov/entrez/query.fcgi?cmd=Retrieve&db=PubMed&dopt=Citation&list_uids=18614008. 10.1016/j.cell.2008.06.030 18614008

[6] Martin A, Orgogozo V (2013) The loci of repeated evolution: A catalog of genetic hotspots of phenotypic variation. Evolution (N Y) 67: 1235–1250. 10.1111/evo.12081 23617905

[7] Stern DL, Orgogozo V (2008) The loci of evolution: how predictable is genetic evolution? Evolution (N Y) 62: 2155–2177. http://www.ncbi.nlm.nih.gov/entrez/query.fcgi?cmd=Retrieve&db=PubMed&dopt=Citation&list_uids=18616572. 10.1111/j.1558-5646.2008.00450.x PMC2613234 18616572

[8] Gompel N, Prud’homme B, Wittkopp PJ, Kassner VA, Carroll SB (2005) Chance caught on the wing: cis-regulatory evolution and the origin of pigment patterns in Drosophila. Nature 433: 481–487. http://www.ncbi.nlm.nih.gov/entrez/query.fcgi?cmd=Retrieve&db=PubMed&dopt=Citation&list_uids=15690032. 15690032 10.1038/nature03235

[9] Chan YF, Marks ME, Jones FC, Villarreal G Jr., Shapiro MD, et al (2010) Adaptive evolution of pelvic reduction in sticklebacks by recurrent deletion of a Pitx1 enhancer. Science (80-) 327: 302–305. http://www.ncbi.nlm.nih.gov/entrez/query.fcgi?cmd=Retrieve&db=PubMed&dopt=Citation&list_uids=20007865. 20007865 10.1126/science.1182213 PMC3109066

[10] Frankel N, Erezyilmaz DF, McGregor AP, Wang S, Payre F, et al (2011) Morphological evolution caused by many subtle-effect substitutions in regulatory DNA. Nature 474: 598–603. http://www.ncbi.nlm.nih.gov/entrez/query.fcgi?cmd=Retrieve&db=PubMed&dopt=Citation&list_uids=21720363. 10.1038/nature10200 21720363 PMC3170772

[11] Reed RD, Papa R, Martin A, Hines HM, Counterman BA, et al (2011) optix drives the repeated convergent evolution of butterfly wing pattern mimicry. Science 333: 1137–1141. 10.1126/science.1208227 21778360

[12] Studer A, Zhao Q, Ross-Ibarra J, Doebley J (2011) Identification of a functional transposon insertion in the maize domestication gene tb1. Nat Genet 43: 1160–1163. Available: 10.1038/ng.942 21946354 PMC3686474

[13] McGregor AP, Orgogozo V, Delon I, Zanet J, Srinivasan DG, et al (2007) Morphological evolution through multiple cis-regulatory mutations at a single gene. Nature 448: 587–590. http://www.ncbi.nlm.nih.gov/entrez/query.fcgi?cmd=Retrieve&db=PubMed&dopt=Citation&list_uids=17632547. 17632547 10.1038/nature05988

[14] Frankel N, Davis GK, Vargas D, Wang S, Payre F, et al (2010) Phenotypic robustness conferred by apparently redundant transcriptional enhancers. Nature 466: 490–493. 10.1038/nature09158 20512118 PMC2909378

[15] Williams TM, Selegue JE, Werner T, Gompel N, Kopp A, et al (2008) The regulation and evolution of a genetic switch controlling sexually dimorphic traits in Drosophila. Cell 134: 610–623. Available: http://www.ncbi.nlm.nih.gov/entrez/query.fcgi?cmd=Retrieve&db=PubMed&dopt=Citation&list_uids=18724934. 10.1016/j.cell.2008.06.052 18724934 PMC2597198

[16] Jeong S, Rokas A, Carroll SB (2006) Regulation of body pigmentation by the Abdominal-B Hox protein and its gain and loss in Drosophila evolution. Cell 125: 1387–1399. http://www.ncbi.nlm.nih.gov/entrez/query.fcgi?cmd=Retrieve&db=PubMed&dopt=Citation&list_uids=16814723. 16814723 10.1016/j.cell.2006.04.043

[17] Jeong S, Rebeiz M, Andolfatto P, Werner T, True J, et al (2008) The evolution of gene regulation underlies a morphological difference between two Drosophila sister species. Cell 132: 783–793. http://www.ncbi.nlm.nih.gov/entrez/query.fcgi?cmd=Retrieve&db=PubMed&dopt=Citation&list_uids=18329365. 10.1016/j.cell.2008.01.014 18329365

[18] Rebeiz M, Pool JE, Kassner VA, Aquadro CF, Carroll SB (2009) Stepwise modification of a modular enhancer underlies adaptation in a Drosophila population. Science (80-) 326: 1663–1667. http://www.ncbi.nlm.nih.gov/entrez/query.fcgi?cmd=Retrieve&db=PubMed&dopt=Citation&list_uids=20019281. 20019281 10.1126/science.1178357 PMC3363996

[19] Wittkopp PJ, Vaccaro K, Carroll SB (2002) Evolution of yellow gene regulation and pigmentation in Drosophila. Curr Biol 12: 1547–1556. http://www.ncbi.nlm.nih.gov/entrez/query.fcgi?cmd=Retrieve&db=PubMed&dopt=Citation&list_uids=12372246. 12372246 10.1016/s0960-9822(02)01113-2

[20] Rogers W a, Salomone JR, Tacy DJ, Camino EM, Davis K a, et al (2013) Recurrent modification of a conserved cis-regulatory element underlies fruit fly pigmentation diversity. PLoS Genet 9: e1003740 http://www.pubmedcentral.nih.gov/articlerender.fcgi?artid=3757066&tool=pmcentrez&rendertype=abstract. 10.1371/journal.pgen.1003740 24009528 PMC3757066

[21] Ordway AJ, Hancuch KN, Johnson W, Wiliams TM, Rebeiz M (2014) The expansion of body coloration involves coordinated evolution in cis and trans within the pigmentation regulatory network of Drosophila prostipennis. Dev Biol: 1–10. 10.1016/j.ydbio.2014.05.023 24907418

[22] Wittkopp PJ, Carroll SB, Kopp A (2003) Evolution in black and white: genetic control of pigment patterns in Drosophila. Trends Genet 19: 495–504. http://www.ncbi.nlm.nih.gov/entrez/query.fcgi?cmd=Retrieve&db=PubMed&dopt=Citation&list_uids=12957543. 12957543 10.1016/S0168-9525(03)00194-X

[23] Keller I, Alexander JM, Holderegger R, Edwards PJ (2013) Widespread phenotypic and genetic divergence along altitudinal gradients in animals. J Evol Biol 26: 2527–2543. http://www.ncbi.nlm.nih.gov/pubmed/24128377. Accessed 15 September 2014. 10.1111/jeb.12255 24128377

[24] Lee T (1963) Genetic analysis of the polymorphism of color pattern in D. auraria. Drosoph Inf Serv 37: 97–98.

[25] True JR, Yeh S-D, Hovemann BT, Kemme T, Meinertzhagen IA, et al (2005) Drosophila tan Encodes a Novel Hydrolase Required in Pigmentation and Vision. PLoS Genet 1: 12 http://www.pubmedcentral.nih.gov/articlerender.fcgi?artid=1285064&tool=pmcentrez&rendertype=abstract. 10.1371/journal.pgen.0010063 PMC1285064 16299587

[26] Wright TR (1987) The genetics of biogenic amine metabolism, sclerotization, and melanization in Drosophila melanogaster. Adv Genet 24: 127–222. Available: http://www.ncbi.nlm.nih.gov/pubmed/3124532. Accessed 3 August 2014. 3124532

[27] Hovemann BT, Ryseck RP, Walldorf U, Stortkuhl KF, Dietzel ID, et al (1998) The Drosophila ebony gene is closely related to microbial peptide synthetases and shows specific cuticle and nervous system expression. Gene 221: 1–9. http://www.ncbi.nlm.nih.gov/entrez/query.fcgi?cmd=Retrieve&db=PubMed&dopt=Citation&list_uids=9852943. 9852943 10.1016/s0378-1119(98)00440-5

[28] Wittkopp PJ, True JR, Carroll SB (2002) Reciprocal functions of the Drosophila yellow and ebony proteins in the development and evolution of pigment patterns. Development 129: 1849–1858. http://www.ncbi.nlm.nih.gov/entrez/query.fcgi?cmd=Retrieve&db=PubMed&dopt=Citation&list_uids=11934851. 11934851 10.1242/dev.129.8.1849

[29] Rebeiz M, Williams TM (2011) Experimental approaches to evaluate the contributions of candidate cis-regulatory mutations to phenotypic evolution. Methods Mol Biol 772: 351–375. Available: http://www.ncbi.nlm.nih.gov/pubmed/22065449. 10.1007/978-1-61779-228-1_21 22065449

[30] Olson M V (1999) When less is more: gene loss as an engine of evolutionary change. Am J Hum Genet 64: 18–23. http://www.pubmedcentral.nih.gov/articlerender.fcgi?artid=1377697&tool=pmcentrez&rendertype=abstract. Accessed 7 September 2014. 9915938 10.1086/302219 PMC1377697

[31] Wittkopp PJ, Stewart EE, Arnold LL, Neidert AH, Haerum BK, et al (2009) Intraspecific polymorphism to interspecific divergence: genetics of pigmentation in Drosophila. Science 326: 540–544. http://www.ncbi.nlm.nih.gov/pubmed/19900891. Accessed 30 July 2014. 10.1126/science.1176980 19900891

[32] Kopp A, Graze RM, Xu S, Carroll SB, Nuzhdin S V (2003) Quantitative trait loci responsible for variation in sexually dimorphic traits in Drosophila melanogaster. Genetics 163: 771–787. http://www.ncbi.nlm.nih.gov/entrez/query.fcgi?cmd=Retrieve&db=PubMed&dopt=Citation&list_uids=12618413. 12618413 10.1093/genetics/163.2.771 PMC1462463

[33] Watt WB (1969) ADAPTIVE SIGNIFICANCE OF PIGMENT POLYMORPHISMS IN COLIAS BUTTERFLIES, II. THERMOREGULATION AND PHOTOPERIODICALLY CONTROLLED MELANIN VARIATION IN Colias eurytheme. Proc Natl Acad Sci U S A 63: 767–774. http://www.pubmedcentral.nih.gov/articlerender.fcgi?artid=223518&tool=pmcentrez&rendertype=abstract. Accessed 24 September 2014. 16591777 10.1073/pnas.63.3.767 PMC223518

[34] Ramniwas S, Kajla B, Dev K, Parkash R (2013) Direct and correlated responses to laboratory selection for body melanisation in Drosophila melanogaster: support for the melanisation-desiccation resistance hypothesis. J Exp Biol 216: 1244–1254. http://www.ncbi.nlm.nih.gov/pubmed/23239892. Accessed 24 September 2014. 10.1242/jeb.076166 23239892

[35] Brisson JA, De Toni DC, Duncan I, Templeton AR (2005) Abdominal pigmentation variation in drosophila polymorpha: geographic variation in the trait, and underlying phylogeography. Evolution 59: 1046–1059. http://www.ncbi.nlm.nih.gov/pubmed/16136804. Accessed 24 September 2014. 16136804

[36] Bastide H, Yassin A, Johanning EJ, Pool JE (2014) Pigmentation in Drosophila melanogaster reaches its maximum in Ethiopia and correlates most strongly with ultra-violet radiation in sub-Saharan Africa. BMC Evol Biol 14: 179 http://www.ncbi.nlm.nih.gov/pubmed/25115161. Accessed 14 August 2014. 10.1186/s12862-014-0179-y 25115161 PMC4236528

[37] Wittkopp PJ, Smith-Winberry G, Arnold LL, Thompson EM, Cooley AM, et al (2011) Local adaptation for body color in Drosophila americana. Heredity (Edinb) 106: 592–602. http://www.pubmedcentral.nih.gov/articlerender.fcgi?artid=3183901&tool=pmcentrez&rendertype=abstract. Accessed 24 September 2014. 20606690 10.1038/hdy.2010.90 PMC3183901

[38] Matute DR, Harris A (2013) the Influence of Abdominal Pigmentation on Desiccation and Ultraviolet Resistance in Two Species of Drosophila. Evolution (N Y): n/a–n/a. 10.1111/evo.12122 Accessed 18 May 2013. 23888866

[39] Parkash R, Rajpurohit S, Ramniwas S (2008) Changes in body melanisation and desiccation resistance in highland vs. lowland populations of D. melanogaster. J Insect Physiol 54: 1050–1056. http://www.ncbi.nlm.nih.gov/entrez/query.fcgi?cmd=Retrieve&db=PubMed&dopt=Citation&list_uids=18519137. 10.1016/j.jinsphys.2008.04.008 18519137

[40] Munjal A, Karan D, Gibert P, Moreteau B, Parkash R, et al (1997) Thoracic trident pigmentation in Drosophila melanogaster: latitudinal and altitudinal clines in Indian populations. Genet Sel Evol 29: 601–610.

[41] Rogers W a, Grover S, Stringer SJ, Parks J, Rebeiz M, et al (2014) A survey of the trans-regulatory landscape for Drosophila melanogaster abdominal pigmentation. Dev Biol 385: 417–432. http://www.ncbi.nlm.nih.gov/pubmed/24269556. Accessed 11 January 2014. 10.1016/j.ydbio.2013.11.013 24269556

[42] Gompel N, Prud’homme B (2009) The causes of repeated genetic evolution. Dev Biol 332: 36–47. http://www.ncbi.nlm.nih.gov/entrez/query.fcgi?cmd=Retrieve&db=PubMed&dopt=Citation&list_uids=19433086. 10.1016/j.ydbio.2009.04.040 19433086

[43] Prud’homme B, Gompel N, Rokas A, Kassner VA, Williams TM, et al (2006) Repeated morphological evolution through cis-regulatory changes in a pleiotropic gene. Nature 440: 1050–1053. http://www.ncbi.nlm.nih.gov/entrez/query.fcgi?cmd=Retrieve&db=PubMed&dopt=Citation&list_uids=16625197. 16625197 10.1038/nature04597

[44] Wittkopp PJ, Williams BL, Selegue JE, Carroll SB (2003) Drosophila pigmentation evolution: divergent genotypes underlying convergent phenotypes. Proc Natl Acad Sci U S A 100: 1808–1813. http://www.ncbi.nlm.nih.gov/entrez/query.fcgi?cmd=Retrieve&db=PubMed&dopt=Citation&list_uids=12574518. 12574518 10.1073/pnas.0336368100 PMC149915

[45] Prud’homme B, Gompel N, Carroll SB (2007) Emerging principles of regulatory evolution. Proc Natl Acad Sci U S A 104 Suppl: 8605–8612. http://www.ncbi.nlm.nih.gov/entrez/query.fcgi?cmd=Retrieve&db=PubMed&dopt=Citation&list_uids=17494759. 17494759 10.1073/pnas.0700488104 PMC1876436

[46] Frankel N, Wang S, Stern DL (2012) Conserved regulatory architecture underlies parallel genetic changes and convergent phenotypic evolution. Proc Natl Acad Sci. Available: http://www.pnas.org/content/early/2012/11/28/1207715109.abstract. 10.1073/pnas.1207715109 PMC3529038 23197832

[47] Laimins L, Holmgren-König M, Khoury G (1986) Transcriptional “silencer” element in rat repetitive sequences associated with the rat insulin 1 gene locus. Proc Natl Acad Sci U S A 83: 3151–3155. http://www.pubmedcentral.nih.gov/articlerender.fcgi?artid=323470&tool=pmcentrez&rendertype=abstract. Accessed 29 September 2014. 3010279 10.1073/pnas.83.10.3151 PMC323470

[48] Udvardy A, Maine E, Schedl P (1985) The 87A7 chromomere. Identification of novel chromatin structures flanking the heat shock locus that may define the boundaries of higher order domains. J Mol Biol 185: 341–358. http://www.ncbi.nlm.nih.gov/pubmed/2997449. Accessed 29 September 2014. 2997449 10.1016/0022-2836(85)90408-5

[49] Burgess-Beusse B, Farrell C, Gaszner M, Litt M, Mutskov V, et al (2002) The insulation of genes from external enhancers and silencing chromatin. Proc Natl Acad Sci U S A 99 Suppl 4: 16433–16437. http://www.ncbi.nlm.nih.gov/entrez/query.fcgi?cmd=Retrieve&db=PubMed&dopt=Citation&list_uids=12154228. 12154228 10.1073/pnas.162342499 PMC139905

[50] Akbari OS, Bae E, Johnsen H, Villaluz A, Wong D, et al (2008) A novel promoter-tethering element regulates enhancer-driven gene expression at the bithorax complex in the Drosophila embryo. Development 135: 123–131. http://www.ncbi.nlm.nih.gov/entrez/query.fcgi?cmd=Retrieve&db=PubMed&dopt=Citation&list_uids=18045839. 18045839 10.1242/dev.010744 PMC2205987

[51] Calhoun VC, Stathopoulos A, Levine M (2002) Promoter-proximal tethering elements regulate enhancer-promoter specificity in the Drosophila Antennapedia complex. Proc Natl Acad Sci U S A 99: 9243–9247. http://www.ncbi.nlm.nih.gov/entrez/query.fcgi?cmd=Retrieve&db=PubMed&dopt=Citation&list_uids=12093913. 12093913 10.1073/pnas.142291299 PMC123125

[52] Frankel N (2012) Multiple layers of complexity in cis-regulatory regions of developmental genes. Dev Dyn 241: 1857–1866. http://www.ncbi.nlm.nih.gov/pubmed/22972751. Accessed 16 September 2014. 10.1002/dvdy.23871 22972751

[53] Barolo S (2011) Shadow enhancers: Frequently asked questions about distributed cis-regulatory information and enhancer redundancy. BioEssays news Rev Mol Cell Dev Biol 34: 135–141. http://www.ncbi.nlm.nih.gov/pubmed/22083793. 10.1002/bies.201100121 PMC3517143 22083793

[54] Schneider CA, Rasband WS, Eliceiri KW (2012) NIH Image to ImageJ: 25 years of image analysis. Nat Methods 9: 671–675. http://www.ncbi.nlm.nih.gov/pubmed/22930834. Accessed 17 July 2014. 22930834 10.1038/nmeth.2089 PMC5554542

[55] Bischof J, Maeda RK, Hediger M, Karch F, Basler K (2007) An optimized transgenesis system for Drosophila using germ-line-specific phiC31 integrases. Proc Natl Acad Sci U S A 104: 3312–3317. http://www.ncbi.nlm.nih.gov/entrez/query.fcgi?cmd=Retrieve&db=PubMed&dopt=Citation&list_uids=17360644. 17360644 10.1073/pnas.0611511104 PMC1805588

