# Supplementary figures and images for: Genetic Changes to a Transcriptional Silencer Element Confers Phenotypic Diversity within and between Drosophila Species
Source: PLoS Genet. 2015 Jun 26;11(6):e1005279. doi: 10.1371/journal.pgen.1005279 (PMC4483262; doi:10.1371/journal.pgen.1005279)

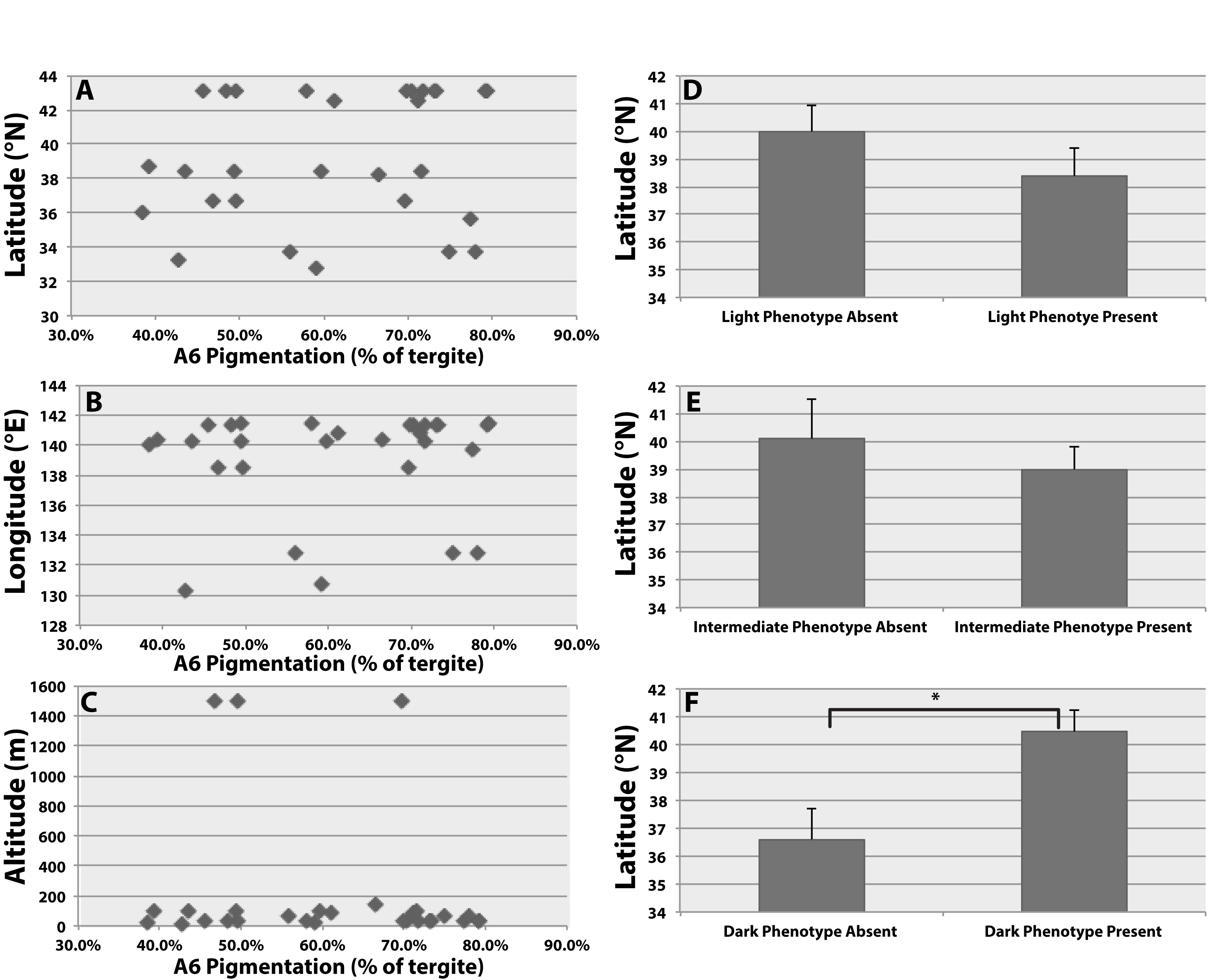

Supplement: S1 Fig — (A-C) Correlation of A6 pigmentation, expressed as the percent of the tergite pigmented, with latitude (A), longitude (B), and altitude (C). (D-F) Analysis latitude, taking into account variation within lines. Isofemale lines were scored based on whether the light (D), intermediate (E), or dark (F) phenotype was absent or present, and the average latitude of these lines were calculated. For the dark phenotype (Panel F), a significant difference in average latitude was detected (* logistical regression: λ2 1 = 7.09, p = 0.0078). (TIF) [file pgen.1005279.s001.tif]

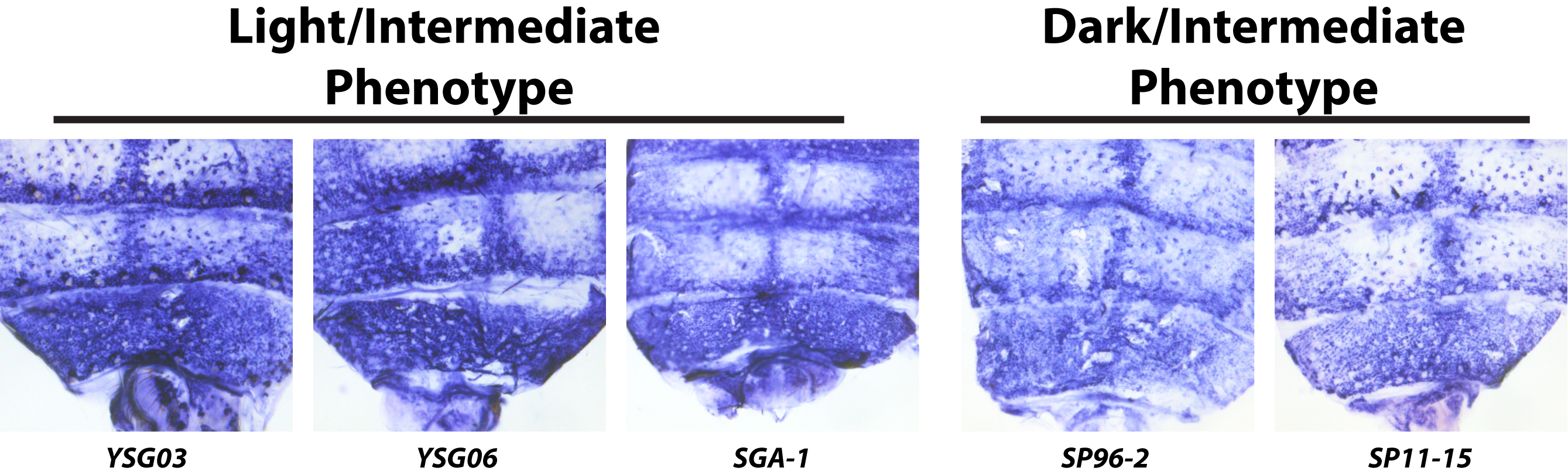

Supplement: S2 Fig — in situ hybridization with a probe to yellow reveals similar patterns of yellow mRNA accumulation between strains containing light (and intermediate) phenotypes compared to those containing dark (and intermediate) phenotypes. (TIF) [file pgen.1005279.s002.tif]

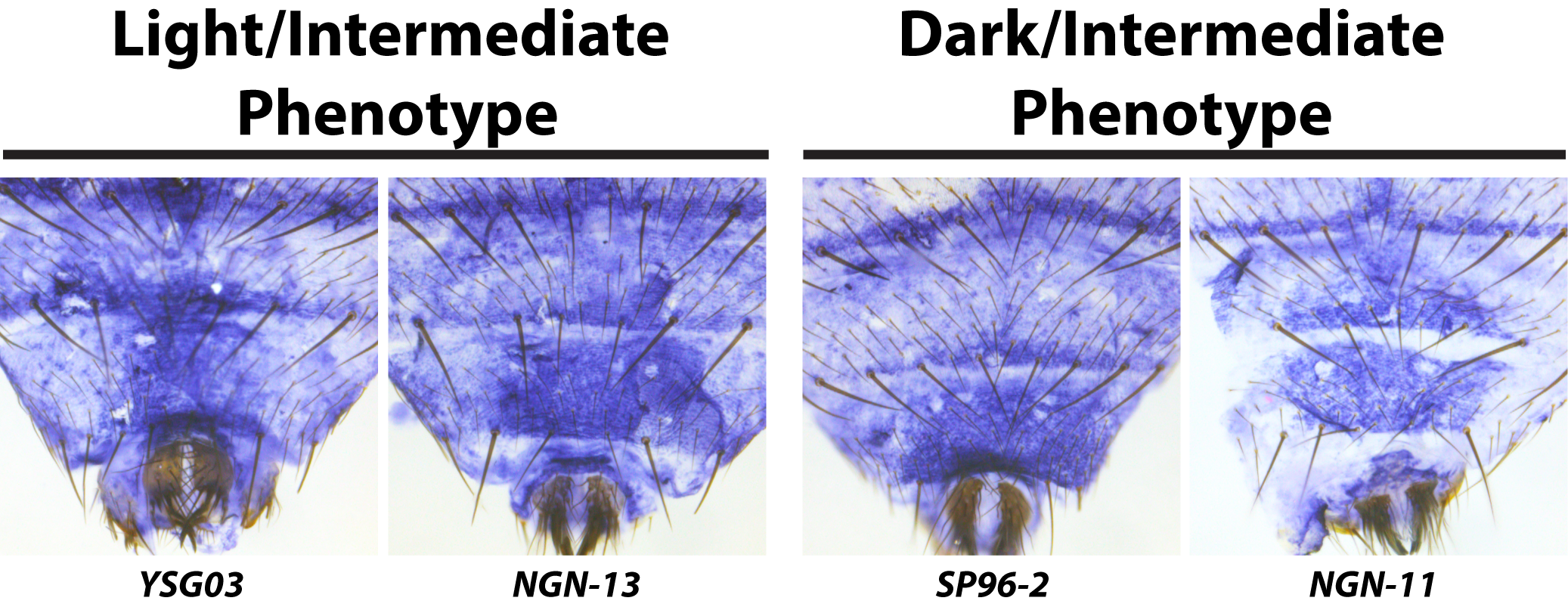

Supplement: S3 Fig — in situ hybridization with a tan probe reveals a similar pattern of mRNA accumulation among strains that contain either the light (and intermediate) or dark (and intermediate) abdominal pigmentation phenotypes. (TIF) [file pgen.1005279.s003.tif]

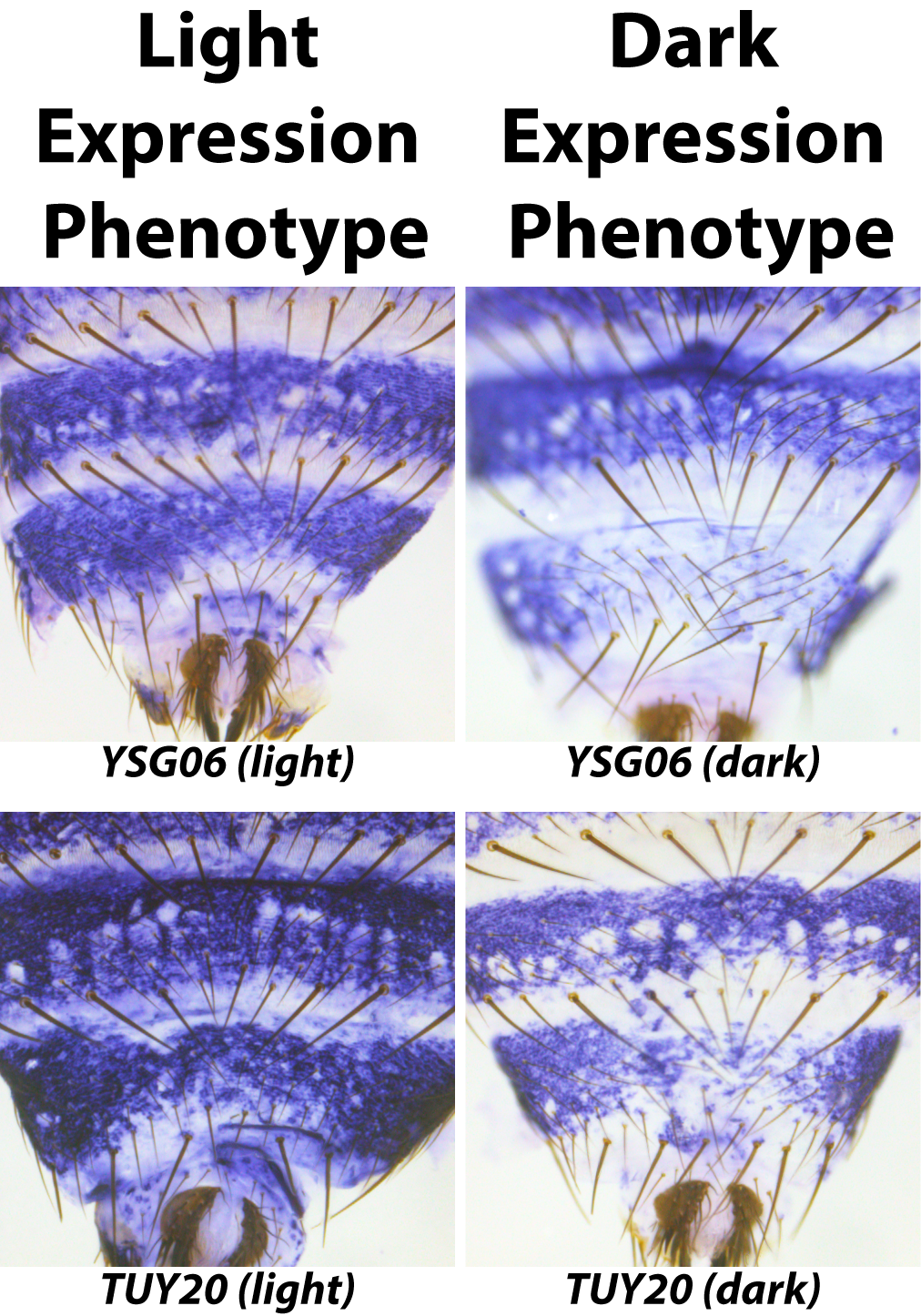

Supplement: S4 Fig — in situ hybridization with an ebony probe reveals lines containing a mixture of ebony expression phenotypes that either match the dark strain phenotype (i.e. reduced midline expression), or the light strain phenotype (uniform expression throughout the anterior portion of the A6 tergite). (TIF) [file pgen.1005279.s004.tif]

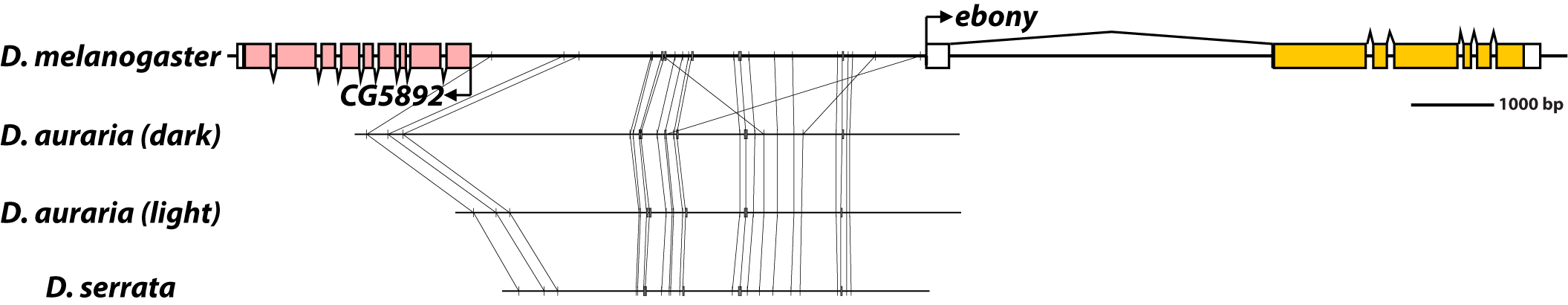

Supplement: S5 Fig — Lines connecting sequences represent conserved sequence blocks of 12 bp or more shared between the species. The difference in length between dark and light auraria strains is caused by a repetitive sequence present in the dark strain sequence. (TIF) [file pgen.1005279.s005.tif]

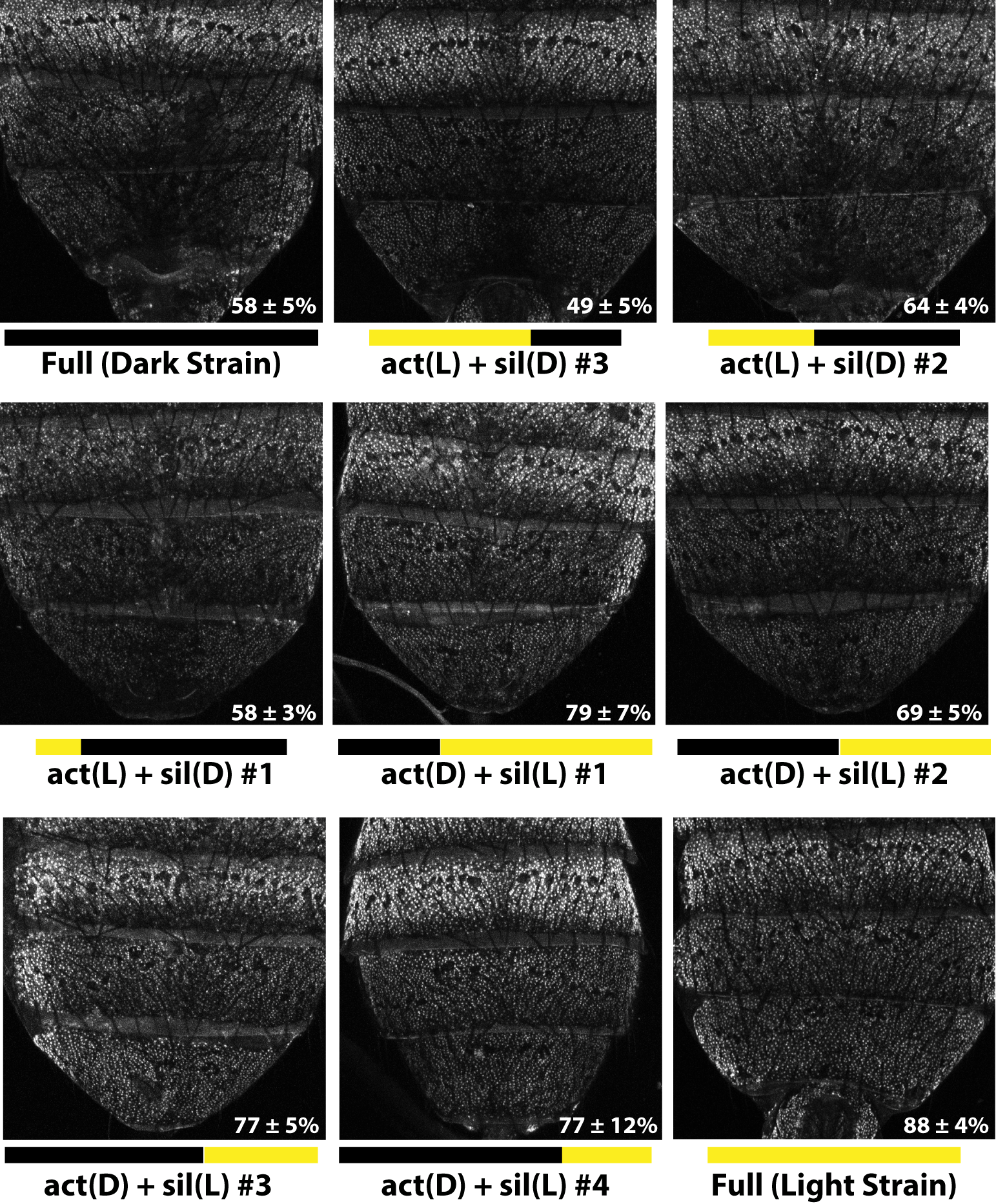

Supplement: S6 Fig — Transgenic reporter lines were imaged 8–10 hours post-eclosion. A6 midline activity ± S.E.M. is shown in the lower-right corner. (TIF) [file pgen.1005279.s006.tif]

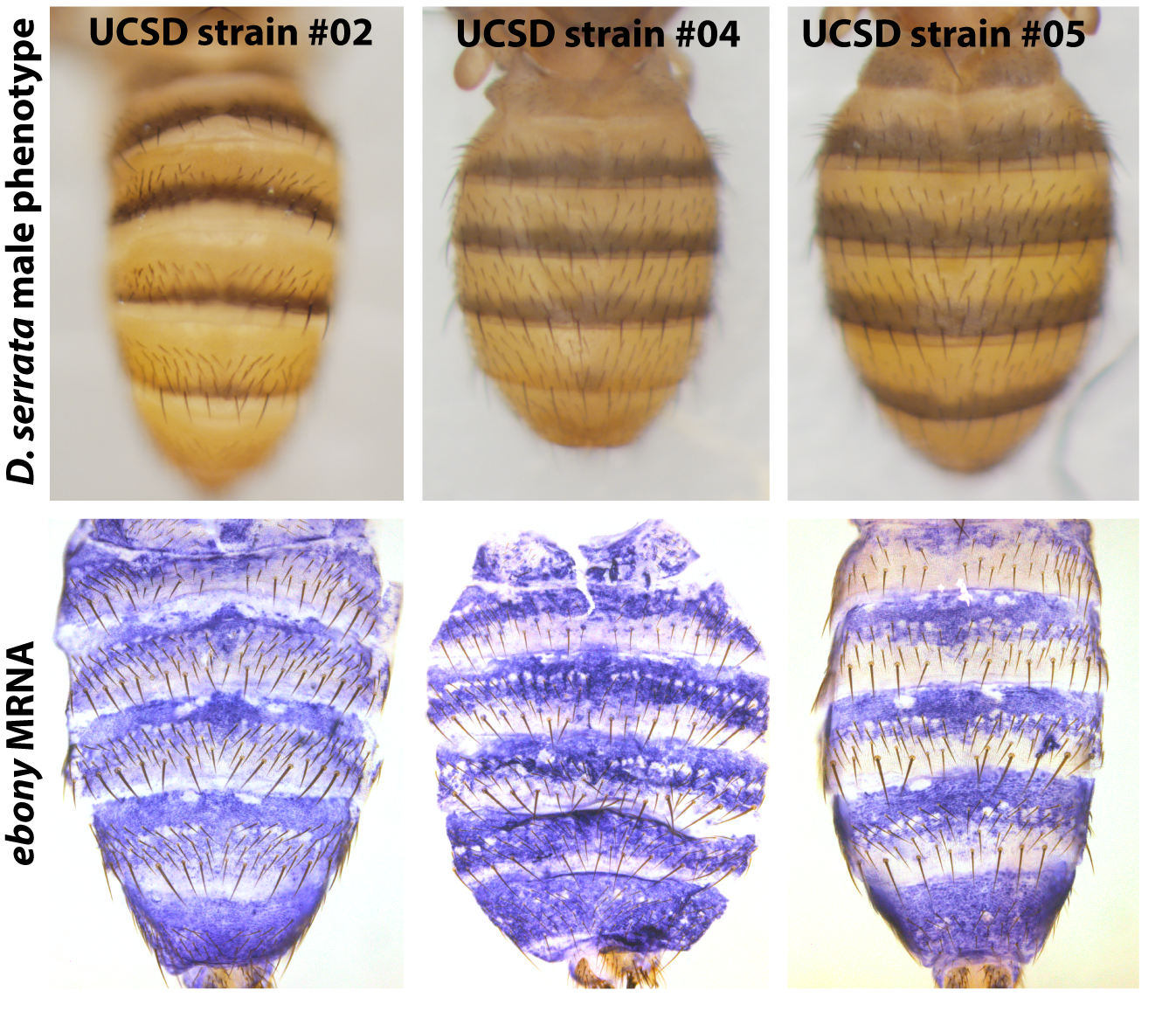

Supplement: S7 Fig — (top) Male phenotypes of D. serrata strains from the UCSD stock center. (bottom) in situ hybridization to ebony reveals gene expression throughout the posterior body segment that has lost pigmentation. Additionally, the extent of ebony mRNA in the posterior portion of each tergite matches pigment stripe phenotype for each strain. (TIF) [file pgen.1005279.s007.tif]
