# Supplementary material for: Genetic Changes to a Transcriptional Silencer Element Confers Phenotypic Diversity within and between Drosophila Species
Source: PLoS Genet. 2015 Jun 26;11(6):e1005279. doi: 10.1371/journal.pgen.1005279 (PMC4483262; doi:10.1371/journal.pgen.1005279)
Supplement: S1 Table — (DOCX) [file pgen.1005279.s008.docx]

| **Species** | **Source** |
| --- | --- |
| *D. auraria "00"* | UCSD: 14028-0471.00 |
| *D. auraria "PM"* | *Mavragani-Tsipidou et al., 1990* (strain #17) [56] |
| *D. serrata "#02"* | UCSD: 14028-0681.02 |
| *D. serrata "#03"* | UCSD: 14028-0681.03 |
| *D. serrata "#04"* | UCSD: 14028-0681.04 |
| *D. serrata "#05"* | UCSD: 14028-0681.05 |
