# Supplementary material for: Genetic Changes to a Transcriptional Silencer Element Confers Phenotypic Diversity within and between Drosophila Species
Source: PLoS Genet. 2015 Jun 26;11(6):e1005279. doi: 10.1371/journal.pgen.1005279 (PMC4483262; doi:10.1371/journal.pgen.1005279)
Supplement: S2 Table — (DOCX) [file pgen.1005279.s009.docx]

| **Species** | **gene** | **Primer F** | **Primer R** |
| --- | --- | --- | --- |
| *D. auraria* | *ebony* | AGCAGCTTCTTCGACTAT | GCTTACAACTAGTCAACA |
| *D. serrata* | *ebony* | GTGCACCGGCTTTACAACTTTTACGG | CAACACAAACTTACGAAACTTCTCGCT |
| *D. auraria* | *tan* | CTGYTGGCCACCAGCAAYGTGGAYG | CATGTGSCGSACATCYTGYTCGCTC |
| *D. auraria* | *yellow* | GGGGAYTGCGCSAACAGYATYACCAC | TGGGRAABAGRTGGGGVCCRCTBG |
