# Supplementary material for: Genetic Changes to a Transcriptional Silencer Element Confers Phenotypic Diversity within and between Drosophila Species
Source: PLoS Genet. 2015 Jun 26;11(6):e1005279. doi: 10.1371/journal.pgen.1005279 (PMC4483262; doi:10.1371/journal.pgen.1005279)
Supplement: S3 Table — Lowercase letters represent appended restriction sites for Asc I (Primer F) and Sbf I (Primer R). Primers for chimeras list the forward and reverse primers used in combination with the D. auraria ebony upstream primers (Primer-F and Primer-R) to generate two or more fragments that were fused together using overlap extension PCR. (DOCX) [file pgen.1005279.s010.docx]

| **Construct** | **Primer-F** | **Primer-R** |
| --- | --- | --- |
| *D. auraria ebony* upstream | TTCCGggcgcgccGCACTTTCGATTAGTCCCATATCC | TTGCCcctgcaggGCTGCAATTATTGCTCGGAGCA |
| *D. serrata ebony* upstream | TTCCGggcgcgccTTNARNCKYTGRTARTCYTG | TTGCCcctgcaggAATTGATCTGTCCTTCGGGATAGG |
| act (L) + sil (D) #1 | GCTACCGCTAATCACTATGAACACGGGTCT | AGACCCGTGTTCATAGTGATTAGCGGTAGC |
| act (L) + sil (D) #2 | GAACAACGTCGATCAGCCGAATGCGAAAGC | GCTTTCGCATTCGGCTGATCGACGTTGTTC |
| act (L) + sil (D) #3 | GATGTCGTAACATTTTGCAGAAGTGAATCACG | CGTGATTCACTTCTGCAAAATGTTACGACATC |
| act (D) + sil (L) #1 | GCTACCGCTAATCACTATGAACACGGGTCT | AGACCCGTGTTCATAGTGATTAGCGGTAGC |
| act (D) + sil (L) #2 | GAACAACGTCGATCAGCCGAATGCGAAAGC | GCTTTCGCATTCGGCTGATCGACGTTGTTC |
| act (D) + sil (L) #3 | TATCGCGTCACAAGCGTGCCTT | AAGGCACGCTTGTGACGCGATA |
| act (D) + sil (L) #4 | GATGTCGTAACATTTTGCAGAAGTGAATCACG | CGTGATTCACTTCTGCAAAATGTTACGACATC |
| CD1 construct | TTCCGggcgcgccGCACTTTCGATTAGTCCCATATCC | TTGCCcctgcaggCTGAGGTAGTGGAACACGAGTA |
| CD2 construct | TTCCGggcgcgccGCACTTTCGATTAGTCCCATATCC | TTGCCcctgcaggAATCATGGGTGTTCCGTGCAAG |
| CD3 construct | TTCCGggcgcgccGCACTTTCGATTAGTCCCATATCC | TTGCCcctgcaggATTCGGCTGATCGACGTTGTTC |
| *D. serrata* chimera breakpoint 1 | GATGTCGTAACATTTTGCAG | CTGCAAAATGTTACGACATC |
| *D. serrata* chimera breakpoint 2 | TCTGATGCTCAGTTCTTATTT | AAATAAGAACTGAGCATCAGA |
